# Identification and Functional Analysis of the CLAVATA3/EMBRYO SURROUNDING REGION (CLE) Gene Family in Wheat

**DOI:** 10.3390/ijms20174319

**Published:** 2019-09-03

**Authors:** Zheng Li, Dan Liu, Yu Xia, Ziliang Li, Na Niu, Shoucai Ma, Junwei Wang, Yulong Song, Gaisheng Zhang

**Affiliations:** College of Agronomy, Northwest A&F University, National Yangling Agricultural Biotechnology & Breeding Center, Yangling Branch of State Wheat Improvement Centre, Wheat Breeding Engineering Research Center, Ministry of Education, Key Laboratory of Crop Heterosis of Shaanxi Province, Yangling 712100, China

**Keywords:** CLE peptide, function analysis, pre-propeptides, mature peptides, wheat

## Abstract

CLAVATA3/EMBRYO SURROUNDING REGION (CLE) peptides are post-translationally cleaved and modified peptides from their corresponding pre-propeptides. Although they are only 12 to 13 amino acids in length, they are important ligands involved in regulating cell proliferation and differentiation in plant shoots, roots, vasculature, and other tissues. They function by interacting with their corresponding receptors. CLE peptides have been studied in many plants, but not in wheat. We identified 104 TaCLE genes in the wheat genome based on a genome-wide scan approach. Most of these genes have homologous copies distributed on sub-genomes A, B, and D. A few genes are derived from tandem duplication and segmental duplication events. Phylogenetic analysis revealed that TaCLE genes can be divided into five different groups. We obtained functional characterization of the peptides based on the evolutionary relationships among the CLE peptide families of wheat, rice, and *Arabidopsis*, and expression pattern analysis. Using chemically synthesized peptides (TaCLE3p and TaCLE34p), we found that TaCLE3 and TaCLE34 play important roles in regulating wheat and *Arabidopsis* root development, and wheat stem development. Overexpression analysis of TaCLE3 in *Arabidopsis* revealed that TaCLE3 not only affects the development of roots and stems, but also affects the development of leaves and fruits. These data represent the first comprehensive information on TaCLE family members.

## 1. Introduction

Since the discovery of systemin, the first plant peptide ligand, proteinaceous ligands have been found to play diverse roles in plant growth, development, and responses to environmental stimuli [1,2]. CLAVATA3/EMBRYO SURROUNDING REGION (CLE) peptide hormones are a group of post-translationally modified proteinaceous ligands involved in the regulation and differentiation of meristematic plant tissues. They control cell division in the shoot apical meristem (SAM), root apical meristem (RAM), vasculature, and legume nodules [3,4,5,6]. CLE peptide hormones belong to a structurally conserved gene family and are named after the first identified CLE peptide (AtCLV3 in *Arabidopsis thaliana*) [7], and the structurally and functionally similar, but unrelated, ESR peptides (first identified in *Zea mays*) [8,9,10].

Mature CLE peptides have a conserved CLE domain composed of 12 to 14 amino acids, which are located at, or near, the C-terminus of their pre-propeptide. CLE pre-propeptides structurally contain a signal peptide at the N-terminus, a variable domain in the middle domain and a conserved CLE domain at the C-terminus [6]. In addition to the typical tripartite domain structure, some also include a fourth domain called a C-terminal extension, which is not highly conserved except between orthologous genes. Some CLE pre-propeptides, containing a multi-CLE domain, have been identified in several plant species, but little is known about their processing in plants [11,12]. There are also a group of CLE-Like (CLEL) peptides that have a functional domain that share a similar structure but exhibit unrelated activity [10].

Members of the CLV3/ESR (CLE) gene family have been found in many angiosperm species. The *Arabidopsis thaliana* CLE family is comprised of at least 32 CLE peptides [13,14], of which CLAVATA3 (CLV3) is the best characterized member. CLV3 controls the fate of stem cells in the shoot apical meristem through the interaction with three major membrane-associated receptor complexes, CLV1, CLV2-CORYNE (CRN),and RECEPTORLIKE PROTEIN KINASE2 (RPK2) [7,15,16,17,18,19]. Among the three receptor complexes, CLV1 and RPK2 are leucine rich repeat receptor-like kinases (LRR-RKs), and CLV2 is an LRR receptor-like protein (LRR-RP) without a kinase domain, but acts together with a membrane-associated protein kinase, CRN, to transmit the CLV3 signal [18,20,21,22]. Mature CLE peptides are probably ligands for LRR receptor-like kinases. A comprehensive list of putative CLE ligand–LRR-RK/RP pairs was presented [23]. The CLV3, CLV14, CLE19, CLV20, and CLE40 peptides all showed an effect on the root meristem through a CLAVATA2 (CLV2)-dependent pathway in *Arabidopsis* [24,25]. Additional *Arabidopsis* CLE peptides acting in the root such as CLE1, 2, 3, 4, and 7 are involved in nitrate- responsive mechanisms, with some also involved in lateral root development [26,27]. AtCLE8 is involved in cell proliferation and differentiation and acts in embryogenesis [28]. AtCLE45 regulates both root protophloem and pollen development [24,29]. AtCLE19, apart from its role in controlling the root meristem, regulates cotyledon establishment, endosperm development and pollen-wall formation in *Arabidopsis* [30,31]. Tracheary Element Differentiation Inhibitory Factor (TDIF), a CLE-like peptide, was originally isolated as a suppressor of tracheary-element differentiation from the Zinnia elegans mesophyll cell-culture medium. The functional TDIF is a 12-AA peptide with two hydroxyproline residues. In *Arabidopsis*, TDIF is identical to the CLE domains of AtCLE41 and AtCLE44, and is highly homologous to those of AtCLE42 and AtCLE46, which determines the fates of procambial cells and controls vascular meristematic tissue proliferation and differentiation by interacting with its receptor PXY/TDR [32,33,34].

In addition to those identified in *Arabidopsis*, a number of CLE peptides have been identified in other plant species including *Lotus japonicus*, *Glycine max*, *Oryza sativa*, *Solanum lycopersicum*, *Pinophyta*, *Populus* and *Physcomitrella patens* [10,12,35,36]. At present, 47 putative CLE genes have been found in the rice genome. The FLORAL ORGAN NUMBER 2 (FON2; also known as FON4) gene, which encodes a CLE protein related to CLV3, is involved as a signaling molecule in a similar pathway to negatively regulate stem cell proliferation in the floral meristem (FM) in rice [37]. The FON1 gene regulates floral meristem size in rice and encodes a leucine-rich repeat receptor kinase orthologous to *Arabidopsis* CLAVATA1 [38]. Because no effect is observed when FON2 is overexpressed in the fon1 mutant, the FON2 signal may be mediated through the FON1 receptor. Thus, the CLV-like signaling pathway appears to be involved in meristem maintenance in grasses. In addition, FON2 SPARE1 (FOS1), encoding another CLE protein, redundantly regulates stem cell maintenance with FON2 in rice [39]. Both FON2-like CLE PROTEIN1 (FCP1) and FCP2 encode proteins with a CLE domain, which are structurally similar to FON2 [40]. However, unlike FON2, which regulates the maintenance of flower and inflorescence meristems, FCP1 regulates the maintenance of the vegetative SAM and RAM.

Bread wheat (*Tricticum aestivum* L.) is an important staple food for humans. Research on the CLE domain proteins in wheat has not been reported, mainly because the genome sequencing of wheat has lagged behind that of other species with the challenges of assembling a large (haploid genome, 1C = 16 Gb), hexaploid, and complex genome that contains more than 85% repetitive DNAs. Fortunately, a fully annotated reference genome of the bread wheat cultivar Chinese Spring (CS) from the Wheat Genome Sequencing Consortium (IWGSC) was recently released. This has promoted the development of rapid and systematic approaches for understanding and selecting important wheat traits. In the present study, we identified 104 TaCLE genes based on the latest released wheat genome. We also studied the potential roles of TaCLE genes involved in the development of stem, root and leaves through the application of CLE peptides in vitro and overexpression analysis of a TaCLE gene in *Arabidopsis*. The results have furthered our understanding of the functions of TaCLE genes in regulating wheat development.

## 2. Results

### 2.1. Identification of CLAVATA3/EMBRYO SURROUNDING (CLE) Peptide-Encoding Genes in Wheat

**Figure 1:**
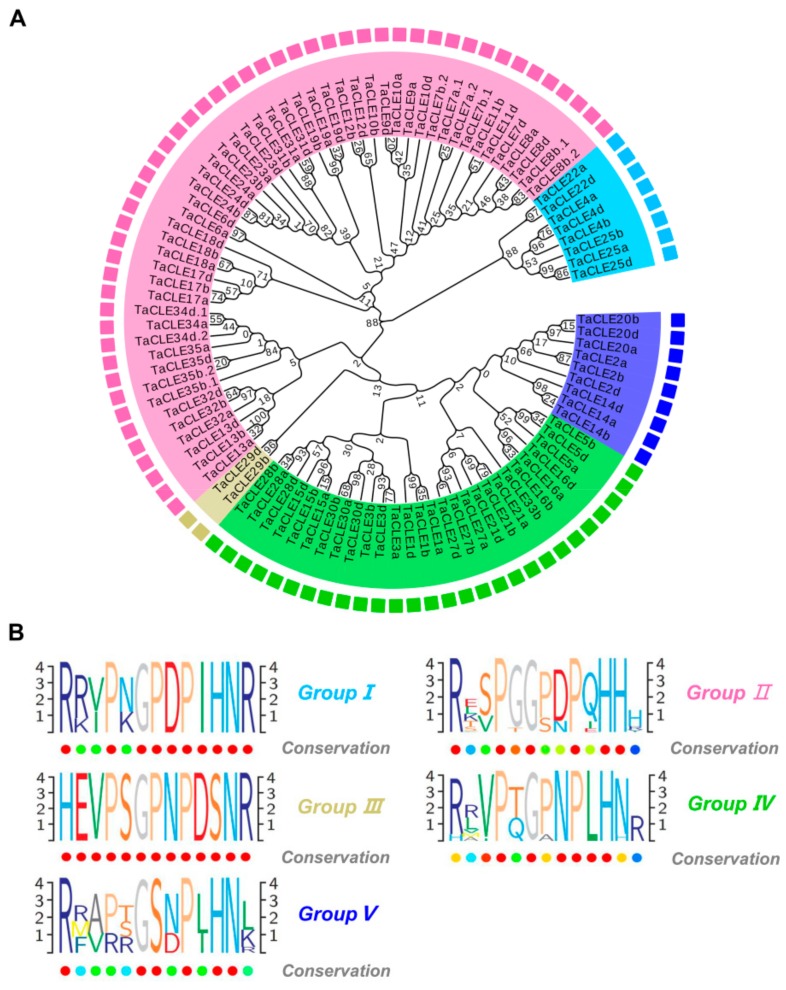
Wheat CLAVATA3/EMBRYO SURROUNDING (CLE) pre-propeptide phylogenetic tree illustrating the five distinct identity groups. (**A**) Phylogenetic analysis was performed using the multiple sequence alignment generated with entire pre-propeptide sequences. Homologous genes consistently clustered together with high confidence (indicated by high bootstrap values). The five groups (Group I–V) were assigned based on clustering in the tree, in addition to sequence similarity. The tree is shown with bootstrap confidence values expressed as a percentage based on 1000 bootstrap replications. (**B**) The CLE domain consensus sequences from the seven wheat pre-propeptide groups. Logo diagrams illustrate the 13 amino acid CLE domain consensus sequences for wheat CLE Groups I–V, determined from multiple sequence alignments generated for each group.

**Figure 2:**
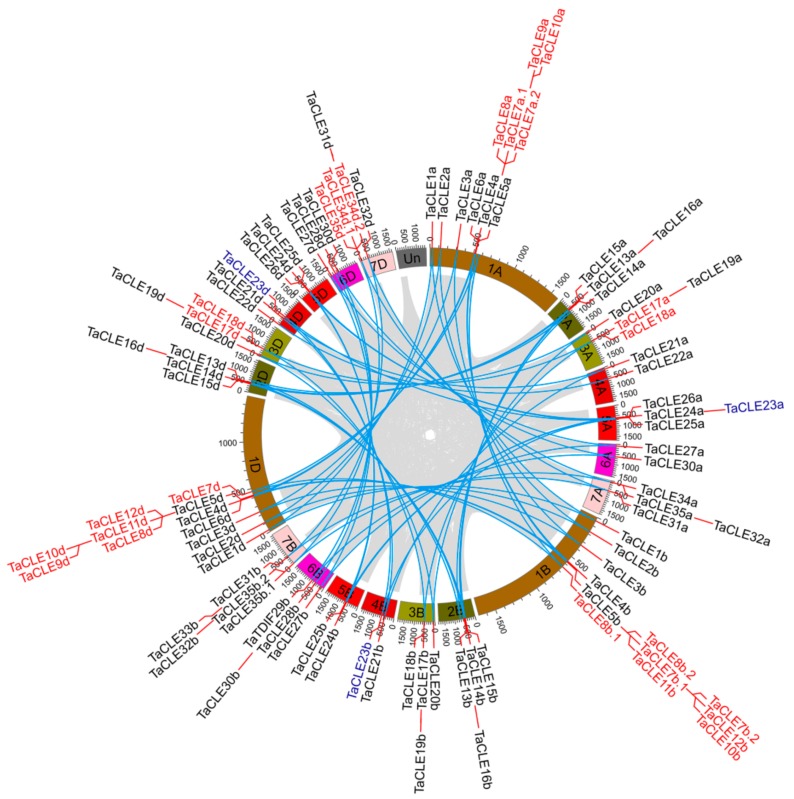
Mapping of duplicated TaCLE genes on wheat chromosomes. Light gray shading represents a collinear relationship among the blocks in whole genome and the deep sky-blue lines represent the collinearity of the TaCLE genes. The red lines indicate that there was a collinear relationship among the tandemly duplicated genes. The red fonts indicate tandem duplication genes. The blue fonts indicate the chromosome distribution of *TaCLE23a*, *TaCLE23b* and *TaCLE23d*.

To identify CLE peptide-encoding genes in wheat, a genome-wide analysis was performed involving multiple BLAST queries and iterative queries, followed by manual validation and the removal of false positives (i.e., no CLE domain). This resulted in the identification of 104 wheat CLE genes (Figure 1A; Supplementary Table S1). Wheat is a heterologous hexaploid plant with three subgenomes A, B and D. Most genes contain highly redundant homologous copies from subgenomes A, B and D (called triplets) in wheat. Apart from triplets, some genes still contain several gene copies due to complex gene-duplication events during wheat evolution. Taking these factors into account, the 104 genes can be integrated into 27 distinct wheat genes. For research convenience these TaCLEs were named *TaARF1–TaARF35* based on their sequence characteristics and their order of distribution on the wheat chromosome in this study (Supplementary Table S1). Of the 104 TaCLE genes identified, most belonged to triplet genes, with some exceptions, such as the absence of *TaCLE6b*, *TaCLE22b*, *TaCLE26b*, *TaCLE29a*, *TaCLE33a*, and *TaCLE33d* (Supplementary Table S1). In this study, we referred to genes with only two homologous copies as twin genes. Therefore, *TaCLE6a,d, TaCLE22a,d, TaCLE26a,d, TaCLE29a,d,* were called twin genes. In addition, *TaCLE23b* and *TaCLE23d* were located on chromosome 4B and chromosome 4D, but *TaCLE23a* was located on chromosome 5A (Figure 2). This may be the result of a segmental duplication event. Although we did not find TaCLE29d in the wheat genomic information database in ensemble plants, the TaCLE29d homologous gene was found in Aegilops tauschii. Considering that Aegilops tauschii is a donor of the wheat D genome, we also named the *TaCLE29d* homologous gene from Aegilops tauschii as *TaCLE29d* and used it in subsequent sequence analysis.

The identified genes were scattered across the genomes with at least three distinct genes located on every chromosome (Figure 2). Most of the identified genes lacked predicted introns, with the exception of 11 wheat genes (Supplementary Table S1), and these identified TaCLE pre-propeptides contained a signal peptide in their N-terminal (Supplementary Figure S1).

### 2.2. Gene Duplication Events in the TaCLE Gene Family

Segmental duplications and multiple genes are created through polyploidy followed by chromosome rearrangements [41]. This occurs most frequently in plants because most plants are diploidized polyploids and retain numerous duplicated chromosomal blocks within their genomes [42]. Tandem duplications are characterized by multiple members of one family occurring within the same intergenic region or in neighboring intergenic regions [42,43]. According to the aforementioned definition, all triple or twin CLE genes described previously were derived from segmental duplication events. Among the named 35 TaCLE genes, *TaCLE7a.1, TaCLE7a.2, TaCLE8a, TaCLE9a* and *TaCLE10a* on chromosome 1A, and *TaCLE7b.1, TaCLE7b.2, TaCLE8b.1, TaCLE8b.2, TaCLE10b, TaCLE11b* and *TaCLE12b* on chromosome 1B, and *TaCLE7d, TaCLE8d, TaCLE9d, TaCLE10d, TaCLE11d* and *TaCLE12d* on chromosome 1D were probably derived from tandem duplication events due to the similarity of their sequences and the proximity of their positions on the chromosome (Figure 2; Supplementary Table S2). Members on different chromosomes were considered to be derived from segmental duplication events. Similarly, *TaCLE17a, TaCLE18a, TaCLE17b, TaCLE18b, TaCLE17d* and *TaCLE18d* located on chromosome 3, and *TaCLE34a, TaCLE35a, TaCLE35b.1, TaCLE35b.2, TaCLE34d.1, TaCLE34d.2* located on chromosome 7 were also considered to be derived from tandem duplication and segmental duplication events (Figure 2; Supplementary Table S2).

### 2.3. Structure Analysis of the TaCLE Gene Family

**Figure 3:**
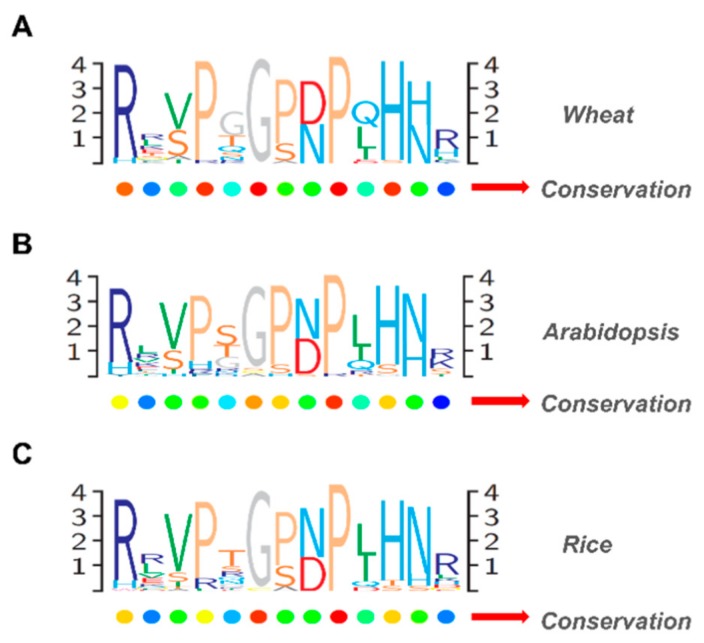
CLAVATA3/EMBRYO SURROUNDING (CLE) domain consensus sequences from wheat pre-propeptides, *Arabidopsis* pre-propeptides, and rice pre-propeptides. Logo diagrams illustrate the 12 or 13 amino acid CLE domain consensus sequences for wheat CLE genes (**A**), *Arabidopsis* CLE genes (**B**), and rice CLE genes (**C**).

Like the structure presented by CLE pre-propeptides in other plants, CLE pre-propeptides in wheat also have a signal peptide at the N-terminus, a central variable domain and a CLE domain at the C-terminus (Supplementary Figure S1). A few genes have a fourth domain called a C-terminal extension. The signal peptide sequences and the variable domain among distinct genes are not highly conserved, except between orthologous genes. For the CLE domain, residues at different positions have different levels of conservation. We compared the CLE domain of all CLE pre-propeptides in wheat, *Arabidopsis* and rice through logo alignments. The results showed that residues 1, 4, 6, 8, 9, 11 and 12 were highly conserved in the wheat CLE domain, which was similar with those of the CLE domain of *Arabidopsis* and rice (Figure 3). This conservation of residues 1, 4, 6, 8, 9, 11 and 12 suggests that they may be critical to the function of TaCLE mature peptides.

### 2.4. Categorization and Functional Predictions of Wheat CLAVATA3/EMBRYO SURROUNDING (CLE) Peptides Based on the Analysis of the Phylogenetic Tree and Expression Patterns

In *Arabidopsis*, CLE peptides are categorized into two groups of A-type and B-type CLE peptides [3]. A-type CLE peptides mainly affect the activity of RAM and SAM, and B-type CLE peptides such as TDIF, affect vascular development [3,44]. The function of many CLE peptides can be predicted based on the sequence characteristics. The wheat CLE peptides were assigned to different categories based on the sequence alignment, phylogenetic analysis, and functional clustering. Five groups (Groups I–V) were identified (Figure 1B). Logo alignments were then constructed to establish the level of conservation within the 13 amino-acid CLE domain. 

Group I consist of eight members (Figure 1A), which shared a similar CLE domain with AtCLE18, AtCLE25, AtCLE26, and AtCLE45 (Supplementary Figure S2). In *Arabidopsis*, CLE25, CLE26 and CLE45 play an important role in regulating root architecture [5,24,45], except for the unknown function of CLE18. Predicted tissue expression showed that the group members may have had high expression in the wheat roots (Supplementary Figure S3). This suggested that the eight members of Group I could also regulate wheat root development. In addition to high expression in the roots, this group also showed possible high expression in stems, leaves and the spikes in the vegetative and reproductive stages, suggesting that the members also regulated the development of multiple tissues or organs. Other residues in the CLE domain of this group were highly conserved, except for residues 2, 3, and 5 (Figure 1B). Residue 5 could either have been asparagine or lysine, which was not available to other group members. Subtle changes in such residues may have contributed to the diversity of the functionality of the mature CLE peptides.

Group II contained 51 members and was the largest group (Figure 1A). These members were predicted to mainly express in roots except for TaCLE13a and TaCLE13b (Supplementary Figure S3). AtCLE1-7 and 40 were assigned to the group (Supplementary Figure S2). AtCLE1, AtCLE4, AtCLE7 and AtCLE40, show specific expression patterns in the root, and are involved in regulating root architecture in *Arabidopsis* [5,46]. In addition, AtCLV3, OsFON2, OsFCP1, and OsFCP2 were also assigned to the group. AtCLV3 and OsFON2 are involved in the maintenance of the reproductive meristems in *Arabidopsis* and rice [7,37]. OsFCP1 and OsFCP2 are involved in the negative regulation of meristem maintenance in the vegetative phase [40]. Therefore, the CLE peptides in Group II not only regulated the maintenance of RAM, but also regulated meristem maintenance in the vegetative or reproductive phases. In addition to these functionally important residues, the residues at other locations showed variation in the CLE domain of the group, which may have led to the diverse functions of the CLE peptides (Figure 1B).

Group III consisted of only three members (Figure 1A), in which TaCLE29 was only identified as TDIF in wheat, unlike three members in rice and four members in *Arabidopsis*. A defining feature of the group is that the CLE peptides begin with a histidine residue, as opposed to the more typical arginine (Supplementary Figure S4). According to evolutionary analysis, TaCLE29 is clustered with *Arabidopsis* CLE41, CLE42, CLE44 and CLE46 and rice CLE102, CLE205 and CLE504 (Supplementary Figure S2). CLE41, CLE42, CLE44 and CLE46 are involved in controlling vascular meristematic tissue proliferation and differentiation in *Arabidopsis* [3]. TaCLE29b showed high predicted expression in all wheat tissues, especially in leaves/shoots and spikes in the vegetative and reproductive stages (Supplementary Figure S3). These results indicated that TaCLE29 may regulate the development of multiple tissues in wheat, and is particularly important for the development of organs in wheat spikes and leaves or shoots.

Group IV consists of 28 members and is the second largest group of TaCLEs (Figure 1A). Residues 4, 6, 7, 8, 9, 10 and 11 showed high conservation in the CLE domains of the group (Figure 1B). Similar to Group III, the CLE domain of TaCLE27 in this group also began with histidine, but TaCLE27 could not be classified as a TDIF-type peptide due to sequence differences (Supplementary Figure S1). Predictive expression patterns indicated that genes in this group were likely to be expressed in various tissues at different stages, unlike Group II (Supplementary Figure S3). This suggested that this group of members regulated the development of multiple tissues in wheat. Some of the wheat CLE pre-propeptides showed similarity to, and grouped closely with, AtCLE 9, 10, 14, 21, and 22 (Supplementary Figure S2). In *Arabidopsis*, CLE9/10 secretory peptide regulates stomatal and vascular development through distinct receptors [47], and CLE14 regulates root development [48,49].

Group V contained nine members (Figure 1A). The CLE domain of the members varied greatly (Figure 1B). In additional to residue 1, 6, 9, 11 and 12, other residues had variation (Figure 1B). In the CLE domain of TaCLE2, the second and third residues were phenylalanine and alanine, respectively, and the third residue was also alanine in the CLE domain of TaCLE20, which differed from the other CLE genes. The fourth residue in the CLE region of TaCLE14 was not a conserved proline, but an arginine, which was also different from other CLE genes (Figure 1B). According to previous studies, the fourth residue has a greater impact on the function of CLE peptides, and its changes seem to contribute to the functional changes of the CLE genes. Phylogenetic tree analysis shows that TaCLE2 and TaCLE20 are closer to OsCLE503 and OsCLE101, but the function of OsCLE503 and OsCLE101 in rice is unknown. AtCLE16, 17 and 20 were also placed into the Group (Supplementary Figure S2). In *Arabidopsis*, AtCLE16 and 17 show functional redundancy for *Arabidopsis* SAM activity [50], and AtCLE17 and 20 can affect root development [51]. Predicted expression patterns indicate that TaCLE2 and TaCLE20 are expressed in various tissues at different stages with a low expression level in roots except for TaCLE2b (Supplementary Figure S3). These data indicate that TaCLE2 and 20 may not only regulate the activity of SAM and RAM, but also regulate other tissues or the development of organs such as stems, leaves and spikes in vegetative and reproductive stages. Curiously, in the construction of a phylogenetic tree according to the mature CLE peptide sequences, TaCLE14 was also assigned to Group I (Supplementary Figure S2). TaCLE14 shares a highly similar CLE domain with that of AtCLE4 and AtCLE45 (Supplementary Figure S2), which indicates that they have the same function.

### 2.5. Multi-CLAVATA3/EMBRYO SURROUNDING Peptide-Encoding Genes of Wheat

Genes encoding pre-propeptides that contained multi-CLE domains were also identified. These included TaCLE26a and TaCLE26d, which had four CLE domains each (Supplementary Figure S5A,B). These four possible CLE domains in the TaCLE26 protein sequence were not identical to each other. These were excluded from the alignment in Figure 1A as they lacked the archetypical domain structure. Evolutionary analysis has demonstrated that TaCLE26 is homologous to AtCLV3, OsCLE502 and OsCLE506 (Supplementary Figure S5A). BothOsCLE502 and OsCLE506 contain multi-CLE domains in rice. However, AtCLV3 does not contain multi-CLE domains in *Arabidopsis*. According to sequence alignment, the first CLE domain of TaCLE26 is highly similar in sequence to the CLE domain of AtCLV3 (Supplementary Figure S5C). Therefore, we speculated that the first CLE domain of TaCLE26 might be the single functional CLE peptide ligand, which was actually translated. 

### 2.6. TaCLE3p and TaCLE34p Affect Wheat Root and Stem Development

According to our classification, Group II and Group VI were the two largest groups in the TaCLE gene family. Most members of the Group II and Group VI showed high expression in the root and stem. Based on the structure analysis of the CLE domain of the TaCLE gene family, the members of the Group II and Group VI were highly conserved in some functional residues. Therefore, we selected a gene TaCLE34 and TaCLE3 from Group II and Group VI as samples for peptide synthesis. In vitro treatment of wheat with the synthetic peptides TaCLE3p and TaCLE34p was an ideal method for simulating the endogenous functions of Group II and Group VI members.

**Figure 4:**
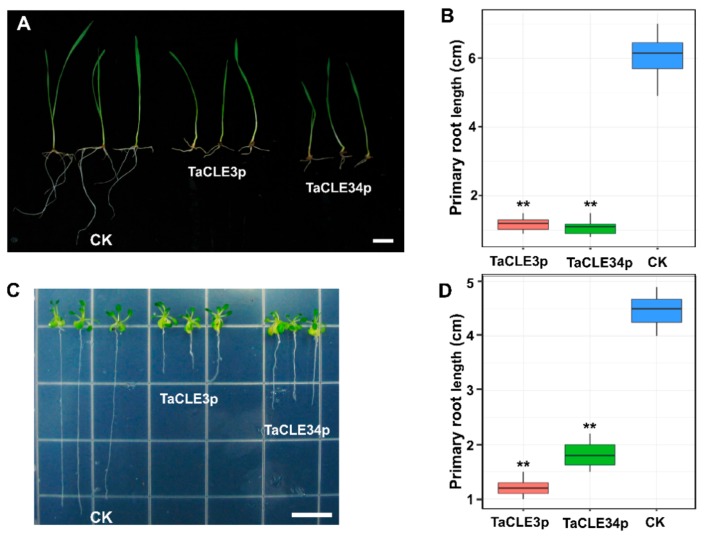
The effect of TaCLE3p and TaCLE34p on wheat and *Arabidopsis* root growth. (**A**,**C**) Representative pictures are shown for wheat (**A**) at 8 d after germination and *Arabidopsis* (**C**) at 12 d after germination. (**B**,**D**) Quantification of the primary root length of wheat seedling (*n* = 10) (**B**), and *Arabidopsis* seedling (*n* = 12) (**D**). The bar graphs indicate the mean ± SE. Statistical significance (Student’s *t*-test) compared with no peptide treatment (CK) is indicated: ** *p* < 0.01. Scale bars: 1 cm in (**A**,**C**).

To investigate the effect of TaCLE3 and TaCLE34 on wheat root development, the primary root length of TaCLE3p-treated and TaCLE34p-treated wheat was analyzed. The length of the primary root of wheat seedlings treated with TaCLE3p and TaCLE34p was significantly shorter than that of the untreated wheat seedlings (Figure 4A,B).

To investigate whether the effect of CLE3p and CLE34p observed in wheat also extended to *Arabidopsis*, the primary root length of CLE3p-treated and CLE34-treated *Arabidopsis* was also analyzed and the results were similar to those in wheat (Figure 4C,D). These results suggest that the orthologues of TaCLE3 and TaCLE34 may also be involved in regulating primary root growth in both monocots and dicots.

**Figure 5:**
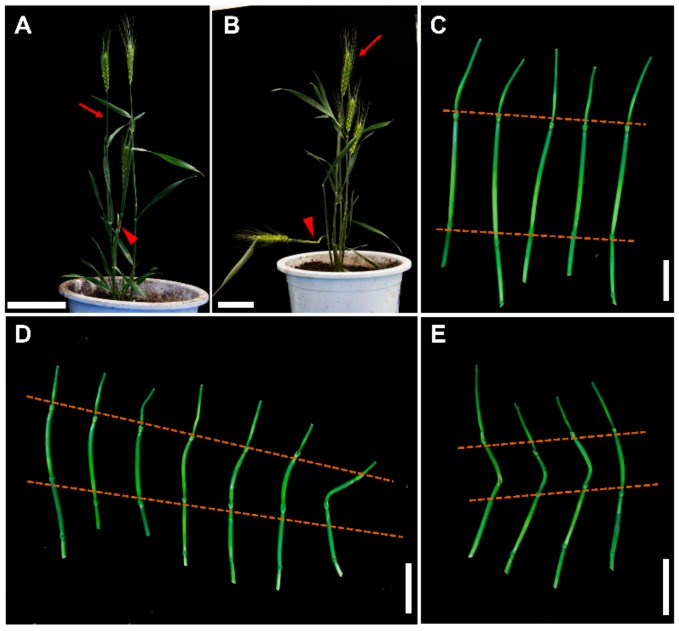
Effect of TaCLE3p and TaCLE34p on wheat stem. (**A**,**B**) Plants at 25 d after TaCLE3p (**A**) and TaCLE34p (**B**) treatment. The red arrows represent the stem of tillers treated with no peptide treatment, and the red triangle symbols represent the main stems treated with the corresponding peptide. (**C**–**E**) The stem of plants 7 d after the treatment of no peptide (**C**), TaCLE3p (**D**) and TaCLE34 (**E**). The red dasheds represent the internodes treated with synthesized peptides. Scale bars: 8 cm in (**A**,**B**), 3 cm in (**C**), 5 cm in (**D**,**B**).

To study the effect of CLE3p and CLE34p on wheat stems, XN1376, a proper height wheat material, was treated with the chemically synthesized peptides at the booting stage. The stems treated with the synthesized peptide were significantly shortened (Figure 5A,B,D,E) and bent 10 d after the end of treatment. The corresponding spike on the treated stem could not be extracted from the flag leaf even at the anthesis stage (Figure 5A,B). This suggested that TaCLE3 and TaCLE34 can affect stem elongation, and exogenously applied TaCLE3p and TaCLE34p inhibit stem elongation.

### 2.7. Overexpression Analysis of TaCLE3d in Arabidopsis

**Figure 6:**
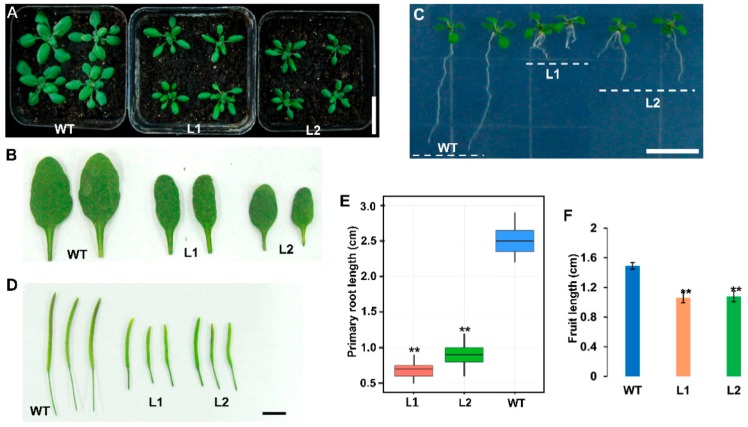
Overexpression of TaCLE3d in *Arabidopsis*. (**A**, **B**) The seedling (**A**) and corresponding leaves (**B**) at 19 d after germination. (**C**) The seedling at 10 d after germination. (D) The fruits from the plants at 35 d after germination. (**E**–**F**) Quantification of the primary root length of the seedling of TaCLE3d-OE (*n* = 10) (**E**), and fruit length (*n* = 18) (**F**). The bar graphs indicate the mean ± SE. Statistical significance (Student’s *t*-test) compared with wild *Arabidopsis* (WT) is indicated: ** *p* < 0.01. Scale bars: 2 cm in (**A**), 1 cm in (**C**), and 0.5 cm in (**D**).

To further evaluate the effect of CLE3p on wheat development and the biological functions of TaCLE3, the TaCLE3 overexpression (TaCLE3d-OE) lines were constructed in *Arabidopsis*. Compared to those of the WT, the leaves of TaCLE3d-OE were thin and smaller, suggesting that the overexpression of TaCLE3d reduced leaf development (Figure 6A,B). Given that TaCLE3 is also expressed in wheat leaves, the result suggests that TaCLE3 is likely involved in regulating development of wheat leaves. Interestingly, the primary root length of TaCLE3d-OE was significantly shortened compared to WT (Figure 6C,E). This result was consistent with the results of wheat root and *Arabidopsis* roots treated by CLE3p. The plant height of TaCLE3d-OE was reduced compared to that of the wild type, and there were no lateral branches on the main stem in TaCLE3d-OE, compared to that of WT (Supplementary Figure S6A–C). The reduction of plant height may be related to the alteration of normal stem development. This result was consistent with the results produced by wheat stems treated by CLE3p. In addition to the results above, TaCLE3-OE also reduced *Arabidopsis* pod length compared with WT (Figure 6D,F).

## 3. Discussion

In multicellular organisms, intercellular communication is essential for coordinating growth and differentiation. In higher plants, besides the conventional phytohormones such as auxin, cytokinin, gibberellic acid, abscisic acid, ethylene, and brassinosteroids, CLE peptides also play an important role in mediating cell-to-cell signaling [36]. The CLE genes and peptides in plants are probably ancient, and their CLE domains share a high level of conservation. The conservation of CLE domain sequences is consistent with the multiple fundamental roles that CLE peptides play in plant development. Despite such sequence conservation, CLE genes had not been previously described in wheat. Sequencing projects provide an opportunity for the isolation of these gene families using a genome-wide scan. In this study, we first isolated 104 CLE members based on a fully annotated reference genome. Due to the collinearity of the wheat A, B, and D sub-genomics, many of the 104 genes identified were triplet genes (Figure 2). However, there were some exceptions, such as the lack of one or two such homologous genes. This may be because, during wheat evolution, these homologous genes are absent or became pseudogenes due to insertion, duplication, or deletion events. The wheat CLE members are distributed on each chromosome of wheat. Some TaCLE members are distributed in tandem duplications on the corresponding chromosomes, such as *TaCLE7-12* on chromosome 1, *TaCLE17-18* on chromosome 3, and *TaCLE34-35* on chromosome 7 (Figure 2; Supplementary Table S1). These tandem duplications are considered to be one of the primary driving forces in the evolution of wheat genomes and genetic systems. They provide raw material for the generation of new CLE genes, which, in turn, facilitate the development of new functions [42].

*Arabidopsis* CLE peptides are classified into A-type and B-type based on their effects. A-type peptides usually affect plant root development. Treating plants with the A-type peptides results in arrested root growth [44]. B-type peptides can affect vascular development. Despite this classification, there is potential crosstalk between A- and B-type CLEs. For example, A-type peptides synergistically accelerate B-type peptide-mediated vascular development [44,52]. To compare the functional classification of *Arabidopsis* CLE peptides, the wheat CLE pre-propeptides (excluding multi-CLE domain-encoding genes) were grouped into five distinct categories (Groups I–V) according to phylogenetic analyses of the entire CLE pre-propeptide sequences (Figure 1A) of wheat, and the mature CLE domain sequences of wheat, rice and *Arabidopsis* (Supplementary Figure S2). These classifications helped to clarify the function of TaCLE peptides. In *Arabidopsis*, there are four TDIF peptides, which are classified as B-type CLEs. However, only one TDIF protein (TaCLE29) was identified in wheat. TaCLE29 begins with a histidine residue, which is the same as TDIF peptides in *Arabidopsis* and other plants (Supplementary Figure S4). According to our classification, TaCLE29 belongs to Group III, and Group III also contains four *Arabidopsis* TDIF peptides and two rice TDIF peptides based on the phylogenetic analysis. This classification suggests that TaCLE29 may affect vascular development. In addition, Group II contains the most members of the TaCLE gene, including the aforementioned segmental duplication and tandem duplications of TaCLE members. Although segmental duplication and tandem duplications enrich the number of TaCLE genes, they also introduce functional redundancy for TaCLE during the regulation of wheat tissue development. As an example, the TaCLE genes derived from segmental duplications or tandem duplications share almost identical mature peptide sequences and expression patterns (Supplementary Figure S3). These peptides therefore share the same receptors when regulating root or stem development and likely produce the same effects. The remaining TaCLE members in Group II also showed high levels of expression in roots or stems, except for TaCLE13 (Supplementary Figure S3). According to phylogenetic tree analysis, Group II also includes AtCLE1-7, AtCLE40, AtCLV3, OsFON2, OsFCP1, and OsFCP2 (Supplementary Figure S2). AtCLE1-7 and AtCLE40 can regulate RAM, and affect root development in *Arabidopsis*. This suggests that TaCLE genes belonging to Group II can regulate root development. In addition, Group II can also regulate the maintenance of reproductive meristems and vegetative meristems, similar to the functions of AtCLV3, OsFON2, OsFCP1, and OsFCP2. For example, TaCLE13 exhibits high expression in the wheat spike in the vegetative stage. We also found that in evolutionary relationships, TaCLE13 was closer to OsFCP1, OsFCP2, followed by TaCLE32, TaCLE17, and TaCLE18 (Supplementary Figure S2). These CLE peptides share a highly conserved CLE domain. Based on our classification, these genes are important candidate genes for studying SAM or FM regulation in wheat. Unlike Group II expression patterns, Group I, III, VI, and V are expressed in many different tissues. Although the expression patterns are different, the functions of the members among the five groups are redundant in some tissues such as roots. Most TaCLE genes are detected in the roots with highly expressed transcripts. We also found that most of the *Arabidopsis* homologs contained in each subgroup had root-dependent effects. Therefore, wheat roots are also important tissues for resolving the function of the TaCLE genes. For example, the receptor corresponding to the mature CLE peptides can be further identified in the roots.

In vitro treatment with chemically synthesized CLE peptides is one method for mimicking the function of endogenous CLE peptides. To verify the function of the TaCLE genes we predicted in a grouped format, we selected one TaCLE gene from each of the two groups containing the most members. Synthesized TaCLE3p and TaCLE34p not only caused short roots in wheat (Figure 4A), but also inhibited wheat stem development (Figure 5A–E). According to the expression pattern prediction, TaCLE34 is hardly expressed in stems, but many members of Group II have highly expressed transcripts in stems, and the stem effects obtained by in vitro treatment indicate that this group of conserved CLE motifs is functionally redundant in regulating stem development. TaCLE3 is expressed in both roots and stems, and the phenotype obtained in vitro also reflects its regulation in vivo. Overexpression of TaCLE3 in *Arabidopsis* can also affect root and stem development (Figure 6C; Supplementary Figure S6B). These results confirm the role of TaCLE3 in regulating root and stem development. The development of leaves and fruits is also affected in overexpressing plants. This suggests that additional functions of TaCLE3 remain undiscovered.

By comparing the mature CLE peptide sequences in wheat, rice and *Arabidopsis*, the conserved features of the CLE domain were revealed (Figure 3). In wheat, rice and *Arabidopsis*, the CLE domain had high conservation at residue 6, which was considered to be critical for function. It has a role in enabling the CLE peptide to rotate or bend [6]. Previous research has also shown that a glycine-to-threonine substitution at residue 6 gave the strongest antagonistic effect in the wild type [30]. In addition, the CLE domain also showed high conservation at residues 1, 4, 5, 9 and 11 in wheat, rice and *Arabidopsis*, and the residues are critical for the function of endogenous CLV3 in *Arabidopsis*. In comparison, residues 2, 3, 5, 7 and 13 exhibited low conservation, and these residues are trivial for CLV3 function [53]. Although residue 12 also showed high conservation in wheat, *Arabidopsis* and rice, it did not appear important for CLV3 function. However, it is possible that residue 12 is important for the function of CLE peptides other than CLV3. Therefore, rational residue substitution will be a useful technique for functional dissection of widely occurring TaCLE genes in wheat.

## 4. Materials and Methods

### 4.1. Identification and Characterization Analysis of TaCLE Genes and Gene Expression

To identify the members of the CLE gene family in wheat, all publicly known *Arabidopsis* CLE protein sequences, rice CLE protein sequences, maize CLE protein sequences and the CLE protein sequences of soybean and common bean were used as queries in BLASTP searches against the wheat genome database included in EnsemblPlants (http://plants.ensembl.org) and the NCBI database (https://www.ncbi.nlm.nih.gov/). The retrieved wheat sequences were used as queries to repeat the step in an iterative manner.

Phylogenetic trees were constructed with the program MEGA X (https://www.megasoftware.net/download_form) using the maximum likelihood approach with 1000 bootstrap replicates. All the sequences of *Arabidopsis* CLE genes and rice CLE genes involved in the construction of the phylogenetic tree were collected from the NCBI database and EnsemblPlants database.

Transcriptional data for the meta-analysis was collected from publicly available data sets from the expVIP database against RefSeq1.1 and then viewed as a heat map using R software.

### 4.2. Chromosomal Locations and Gene Duplication

Genes were mapped on chromosomes by identifying their chromosomal position provided in the wheat genome database. Gene duplication was investigated following the method described by [43]. To visualize the duplicated regions in the wheat genome, lines were drawn between matching genes using the Circos tool (http://circos.ca/).

### 4.3. Gene Structure and Protein Conserved Motifs Analysis

Logo diagrams used to define consensus sequences were obtained using multiple sequence alignments for each TaCLE peptide group (I–V), *Arabidopsis* CLE peptides, and rice CLE peptides by TEXshade [54]. Signal peptides were identified using the Simple Modular Architecture Research Tool (SMART) (http://smart.embl-heidelberg.de/) [55].

### 4.4. In Planta Peptide Treatment of Wheat Roots and Stems

Peptides were synthesized by Sangon Biotech (Shanghai, China) with a purity of >95%. Peptide sequences are listed in Supplementary Table S3. The seeds of Chinese Spring (CS) wheat were used as the sample of peptide treatment of wheat roots. Peptide treatment of wheat and *Arabidopsis* roots was performed as previously described [56]. The roots of wheat and *Arabidopsis* were treated with 1 μM concentrations of peptides [56]. The stems of XN1376 were treated with 30 μL peptide (1 μM) in the wheat booting stage. The synthetic peptides were gently injected into the upper end of the second stem section of the main stem using a micro-injector (Hamilton 800, Hamilton Company, Switzerland). Each treatment contained ten independent wheat plants. The second stem section of each individual tiller was injected with 20 μL of sterile water as a control. The same treatment was performed every three days a total of three times. Ten days after the end of the treatment, the treated wheat individuals were observed. Primary root length were measured as previously described [56]. Data was analyzed using Excel to conduct t-tests. Differences with *p* < 0.05 and *p* < 0.01 were considered as significant and extremely significant, respectively. Plant materials were photographed with a Canon EOS7D digital camera (Nikon, Japan). 

### 4.5. Vector Constructs and Plant Transformation, Phenotypic Observation.

Seeds of *Arabidopsis* (ecotype Columbia) were derived from our laboratory. Seeds of *Arabidopsis* were surface-sterilized and sown on 50% MS medium plates. After a 48 h incubation at 4 °C, the plates were transferred to a plant growth incubator (Sanyo, Osaka, Japan) for 7 d before transfer to soil under a 16:8 (L:D) photoperiod at 22 ± 1 °C) in a growth room.

To construct the wheat TaCLE3d gene overexpression vector, the full-length cDNA sequence was PCR amplified and subcloned into pCAMBIA1302 (Supplementary Table S4). The resulting vector was transferred into *Agrobacterium tumefaciens* GV3101 by electroporation and transgenic *Arabidopsis* plants were produced by floral dip [57]. Seeds were harvested and Hygromycin B-resistant first-generation (T1) transgenic plants were screened. Transgenic plants were confirmed by PCR.

## 5. Conclusions

In conclusion, we identified 104 TaCLE members in wheat and categorized them into groups. This provided insight into their structure and function based on key orthologues existing amongst them, *Arabidopsis*, and rice, and transcriptional evidence. Future work is planned to establish the function of these wheat CLE peptides and to identify their receptors and the signaling pathway in which they participate. Determination of the precise structural modifications of the mature peptides is needed to understand how the peptides regulate plant development.

## Supplementary Materials

Supplementary materials can be found at https://www.mdpi.com/1422-0067/20/17/4319/s1.
