# Supplementary material for: Identification and Functional Analysis of the CLAVATA3/EMBRYO SURROUNDING REGION (CLE) Gene Family in Wheat
Source: Int J Mol Sci. 2019 Sep 3;20(17):4319. doi: 10.3390/ijms20174319 (PMC6747155; doi:10.3390/ijms20174319)
Supplement: Supplementary file 1 [file ijms-20-04319-s001.zip › Supplementary Table S3.pdf]

Supplementary Table S1 The information on synthetic peptides

Peptide Name	Sequence	Purity
TaCLE3p	KRLVPQGPNPLHN	98.13%
TaCLE34p	KRSSPGGPDPQHH	96.76%
