# Supplementary material for: Identification and Functional Analysis of the CLAVATA3/EMBRYO SURROUNDING REGION (CLE) Gene Family in Wheat
Source: Int J Mol Sci. 2019 Sep 3;20(17):4319. doi: 10.3390/ijms20174319 (PMC6747155; doi:10.3390/ijms20174319)
Supplement: Supplementary file 1 [file ijms-20-04319-s001.zip › Supplementary Table S4.docx]

| TaCLE3d-OE-F | GGGGGACTCTTGACCATGGAGATGGCGAGATCACGAGACACCA |
| --- | --- |
| TaCLE3d-OE-R | TTCTTCTCCTTTACTAGTTCAGTTGTGCAGTGGGTTCGGC |

**Supplementary Table S2** The primers used in the study
