# Supplementary material for: Identification and Functional Analysis of the CLAVATA3/EMBRYO SURROUNDING REGION (CLE) Gene Family in Wheat
Source: Int J Mol Sci. 2019 Sep 3;20(17):4319. doi: 10.3390/ijms20174319 (PMC6747155; doi:10.3390/ijms20174319)
Supplement: Supplementary file 1 [file ijms-20-04319-s001.zip › Supplementary Figure S1.pdf]

Signal peptide

```
TaCLE1a      1 --MRRPGGA---------APVVVLLLWLA----------------------------ALTFAFHGCGGG-LLRCGSSVAVVERDRRPASLPRKMLLAVES
TaCLE1d      1 --MRRPGGA---------APVVVLLLWLA----------------------------ALTFAFHGCGGG-LLVRRGSSVAVERDRRPASLPRKMLLAVES
TaCLE1b      1 --MRRPGGA---------APVVVLLLWLT----------------------------ALTFAFHGCGGGGLLRCGG--LAVQLD-HPASLPRKMLLVVES
TaCLE15a     1 -MVRDTGGA--------LQL-LAVLLLVL----------------------------ASELATFSCGHR--------------------IPRADVAAWK-
TaCLE15b     1 -MVRDTGGA--------LQL-LAVLLLLL----------------------------ASELATFSCGHR--------------------IPRADVAAWK-
TaCLE15d     1 -MVRDTGGA--------LHL-LAVLLLLL----------------------------ASELATFSCGHR--------------------IPRADVAAWK-
TaCLE28b     1 -MRRRAGGAGTPPLPDTLSVSLLLLLLLV----------------------------ATELGTAACVCG--------------------VLKADAVAAA-
TaCLE28d     1 -MRRRAGGAGAPPLPDTLSVSLLLLLLLV----------------------------ATELGTAACVCG--------------------VLKADAVASA-
TaCLE3b      1 -MARSRDT----------RLRLTLLLVAL----------------------------LLLVDMASVCHG------------------RRTP--DVDAM--
TaCLE3d      1 -MARSRDT----------RLRLTLLLVAL----------------------------LLLVDMASVSHG------------------RRTP--DVDAM--
TaCLE3a      1 -MARSRDTR---------LRLRLTLLLVTL----------------------------LLLVDMASVSHG------------------RRTP--DVDAM--
TaCLE30b     1 -MRRPSQQH--------SLLPCILLLLLLL----------------------------VSMSHLPSSSHG------------------LRTLREEEEAVG-
TaCLE30d     1 -MRRSSQQH--------SLLPCILLLMLL----------------------------AMSHLPSSSHG------------------LRTLREEEEAVG-
TaCLE30a     1 -MRRSSQQH--------SLLPCILLLLLL----------------------------VSMSCLPSSSHG------------------LRTLREEEEAVG-
TaCLE5a      1 -MARSWSCA--------LRLLFLASLLLLCSALPPPPPP-GRGSAT----ELTDADEAVLARMCNPRAG------------------PRPAWCEELHLIK
TaCLE5d      1 -MGRSWSCA--------LRLLFLASLLLLCSALPPPPPP-GRGSASASPAELTAADEAVLARMCNPRAG------------------PRPAWCEELHLIR
TaCLE5b      1 -MARSCSCA--------LRLLFLASLLLLCSAPPPPPPPGRGSAS--PAELTAADEAVLARMCNPRAG------------------PRPAWCEELHLIR
TaCLE2a      1 -------MN---------CVKILVLLSLI----------------------------PLALRGASLL---------------------VVPSPSAPDSAS
TaCLE2d      1 -------MN---------CVKILVLLSLI----------------------------PLALRGAALL---------------------VVPSPSSPDSAS
TaCLE2b      1 -------MN---------CVKILVLLSLI----------------------------PLALRGASLL---------------------VVPSPSA-DPAS
TaCLE20a     1 -MGGGVVRC---------CVKILALLSLV----------------------------PLALRAGSLLGH------------------VVAPPPSSARPAA
TaCLE20d     1 -MGGGVVRC---------CVKILALLSLV----------------------------PLALRAGSFLGH------------------VVAPPPSSARPAA
TaCLE20b     1 -MGG-VVR----------CVKILALLSLV----------------------------PLALRAGSLLGH------------------VVAPPPSS----A
TaCLE14b     1 -MRVPSLAL---------AILVLLALSAA----------------------------TGRPAVLAARTP------------------GVLLAPTSSRGVD
TaCLE14d     1 -MRVPSLAL---------AILVLVALSAG----------------------------TSRPGVLAARSP------------------GVLLAPTSSRGVE
TaCLE14a     1 -MRVPSLAL---------AILVLVALSAA----------------------------TGRPGVLAARSP------------------GVLLASTSRRGAE
TaCLE21b     1 ----------------------MVCCSRR----------------------------RDTPAAGGLLGS------------------WLLIAVLVQSSLL
TaCLE21d     1 ----------------------MVCCSRR----------------------------RDRPAAGGLLGS------------------WLLIAVLVQSSLL
TaCLE21a     1 ----------------------MVCCSRR----------------------------RDTPAAGGLLGS------------------CLLIAILVQSSLL
TaCLE16a     1 -MGRQRRWS---------RAALLACLVLL----------------------------AAAACADSARPG------------------PLAAAATEGLPAG
TaCLE16d     1 -MGRQRRWS---------RAALVACLVLL----------------------------AAAACAESARPG------------------PLAAA-TEGLPAG
TaCLE16b     1 -MGRQRRWS---------RAALLACLLLL----------------------------AAA-CAESARPG------------------PLAAA-SEGLPRG
TaCLE27a     1 -MGR-RPLA---------SAAAFVALAVL----------------------------LLVVVVAVHGAG------------------VPSSSVMASVGNG
TaCLE27d     1 -MGR-RPLA---------SAAAFVALAVL----------------------------LLVVVVAVHGAG------------------VPSLSVMASAGNG
TaCLE27b     1 -MGR-RPLA---------SAVAFVALAVL----------------------------LVVVAGAVHGAG------------------VPSSSVMASAGNG
TaCLE33b     1 -MRR-RRWA---------RAAALC-LALA----------------------------VLLLQLAVAHGAG------------------AWAARMPAFSTRA
TaCLE4b      1 -MGRRPDRS-------LGASACCERVVAV----------------------------IFIVCIALLVVM------------------VAMTSGAGVSIGPA
TaCLE4d      1 -MGRRPDRS-------LGASACCERVVAV----------------------------IFIVCIALLLVM------------------VATTSGAGVSLGPA
TaCLE4a      1 -MGRRPDRS-------LGASACCKRVVAV----------------------------IFLVCVALLVVM------------------VATTSGAGVGFGPA
TaCLE22a     1 -MGGIAAGR-------CRISGLALLVCAV----------------------------LLSAT-MANGIR------------------TGAVGGTGAPGPAA
TaCLE22d     1 -MGGIAGGR-------CRISGLALLVCAV----------------------------LLSATTMANGIR------------------TGAADGTGAPGPAA
TaCLE25a     1 MRGPAASGG-------RASAAA--VLFGV----------------------------LVLVSLVVVVAE------------------RPSTPAIRGRRMLL
TaCLE25d     1 MRGPAASGG-------RASAAAPAVLFGV----------------------------LVLVSLVVVVAE------------------RPSAPAIGGRRMLL
TaCLE25b     1 MRGPAASGG-------RASAAA--VLLGV----------------------------LVLVSLVVVVAE------------------RPPAPAIGGRRMLL
TaCLE29b     1 MATARCAGR-------WSAPAL--LLLLL----------------------------LLLCAVALVAVV------------------PLAALPGG------
TaCLE29d     1 MATARCAGG-------WSAPALP-LLLLC----------------------------ALLCAIALVAA------------------PLAAPPGEGTARLP
TaCLE6a      1 ---------------MAAARVG-IVLLVV----------------------------MVIAAQLVATP---------------------------------E
TaCLE6d      1 ---------------MAAARVG-IVLLVV----------------------------MVIAAQLVAAP---------------------------------E
TaCLE17a     1 -----------------MAARIGAAVLCVV----------------------------LIVSLGLIATPT---------------------------------E
TaCLE17b     1 -----------------MAARIGAAVVCLV----------------------------LIVSLGLVATPT---------------------------------E
TaCLE17d     1 -----------------MAARIGAAVLCMV----------------------------LIISAGLVATPT---------------------------------E
TaCLE18a     1 -----------------MAARVGAVVLCML----------------------------LIVSAGLVATPT---------------------------------E
TaCLE18d     1 -----------------MAARVGAVVLCMF----------------------------LIVSAGLVATPT---------------------------------E
TaCLE18b     1 -----------------MAARIGAVVLCMV----------------------------LIVSAGLVATPT---------------------------------E
TaCLE13b     1 -----------------MAHAADARSRCVV----------------------------AVLFAVAVFLAC-------------------LPPAATAS--SS
TaCLE13d     1 -----------------MAHAADARSRCVV----------------------------ALLFAVAVFLAC-------------------LPPAATAS--SS
TaCLE13a     1 -----------------MAHAAHARSRCVV----------------------------ALLFAVAVFLAC-------------------LPPAATASASSS
TaCLE32b     1 -----------------MASSRVAVLLFLV----------------------------CTLGVARKMEAAR------------------MMHKGEVVLGNGG
TaCLE32d     1 -----------------MASSRMAVLLFLV----------------------------CTLGVARKMEAAR------------------MMQKGDVVVGNGG
TaCLE32a     1 -----------------MASLRMAVLLFLV----------------------------CTLGVARKMEAAR------------------MMQRGEVVVGNGG
TaCLE7a.1    1 -------------------MAKACLLRGVG----------------------------LVVCALLLLSPSF------------------MAAEATGRKWDHG
TaCLE7b.1    1 -------------------MAKACILRGVV----------------------------LVVCALLLLSSHF------------------LAAEPAGRQWDHG
TaCLE7a.2    1 -------------------MAKACVLGGVV----------------------------MVVCALMLLSSSF------------------VAAEPGGRQWGHG
TaCLE7b.2    1 -------------------MAKACELRGVL----------------------------MVVCALLLLSSSF------------------VAAEPGGRQWAHG
TaCLE7d      1 -------------------MAKACVLRGAT----------------------------LMVCALLLLSSSF------------------IAAEPAGRQWDHG
TaCLE8b.1    1 -------------------MAKACVLRGAA----------------------------LMVCTLLLLSSSF------------------IVVEPAGRQWDHG
TaCLE8b.2    1 -------------------MAKACVLRCAV----------------------------LMVCTLLLLSSSF------------------MAAETAGRQWDHG
TaCLE8a      1 -------------------MAKACVLRGAM----------------------------LIVCTLLLLSSSF------------------MAAEPAGQQLDHV
TaCLE8d      1 -------------------MAKACVLRGAV----------------------------LMVCALLLLSSAF------------------VAAERAGQQWDNG
TaCLE11b     1 -------------------MAKACVLRRVV----------------------------LVVCVLLLLSSTF------------------VVAEATGGQLDHG
TaCLE11d     1 -----------------MAMAKACVVCRVV----------------------------LVVCALLLLSSTF------------------VVAEATFGQLDHG
TaCLE9a      1 -------------------MAKACVLRGAV----------------------------LVVCALLVLSSAS------------------AAVEAAGRQRGHG
TaCLE9d      1 -------------------MAKACVLRGAV----------------------------LVVCALLVLSSAS------------------ASAEAAGRQSGHG
TaCLE12b     1 -------------------MASACVLRGTV----------------------------LVVCVLLVLSSAS------------------AAREAAGRQRAHG
TaCLE12d     1 -------------------MASACVLRGAV----------------------------LVVCVLLVLSSAS------------------AAAEAAGRQRGHG
TaCLE10b     1 -------------------MAKTRVLRCAV----------------------------LVVCVLLVLSSAS------------------AAAEAAGRQRGHG
TaCLE10a     1 -------------------MAKARVLRCAV----------------------------LVVCALLLLSSAS------------------AAAEAAGRQRGHG
TaCLE10d     1 -------------------MAKARVLRCAV----------------------------LMVCALLVLSS-----------------------EAAGRQRGHG
TaCLE19a     1 -------------------MARTTGLG-------------------------------IFICAVLLLAAAV------------------PLESARVLR----
TaCLE19b     1 -------------------MARTTGLG-------------------------------IFICAVLLLAAAV------------------PLESARVLR----
TaCLE19d     1 -------------------MARTTSLG-------------------------------IFICAVLLLAAAV------------------PSESARVLR----
TaCLE23a     1 ----------------------MRLLLCFC----------------------------ICLVLLL-AGSSP-------------------DPFSERCPLRH-
TaCLE23d     1 ----------------------MRLLLCLC----------------------------ICFVLLL-AGSSP-------------------DSHSERCPLQH-
TaCLE23b     1 ----------------------MRLLLCFC----------------------------IFLVVLL-AGSSS-------------------DSLSERCPLQH-
TaCLE24a     1 ---------------------MTRLLLCFC----------------------------VCFVVLVVAGSSP------------------ADLLSGRCPLHH-
TaCLE24d     1 ---------------------MTRLLPCFC----------------------------VCLVVLLLAGSSP------------------ADPLSGRCPLHHH
TaCLE24b     1 ---------------------MMRLLPCFC----------------------------VCLVVVLLVGSSP------------------ADLLAGRCPLHH-
TaCLE31a     1 ----------------------MKLVMCLC----------------------------IFIVLVI--TSSP-------------------IAVSGDRPLMLG
TaCLE31d     1 ----------------------MKLVMCLC----------------------------IFIVLVI--TASP-------------------IAVSGDRPLMLG
TaCLE31b     1 ----------------------MKLVMCLC----------------------------VFIILVI--ASSP-------------------IPVSGDRPLMLG
TaCLE34a     1 ----------------------MPAAVCAV----------------------------LVLLLS--AVSR------------------CEADLLQVTVAGG
TaCLE34d.1   1 ----------------------MPAAVCAV----------------------------LILLLLSV-AVPC------------------CEADLLQVIVSGG
TaCLE35a     1 ----------------------MP-AVCAV----------------------------LILLLLS-AASR------------------CEADLLQVTVAG-
TaCLE35b.1   1 ----------------------MP-AVCAV----------------------------LILLLLS-AASR------------------CEADLLQVTVAG-
TaCLE35b.2   1 ----------------------MP-AVCAV----------------------------LILLLLS-AASR------------------CKADHLQVTVAG-
TaCLE35d     1 ----------------------MP-AVCAV----------------------------LILLLLS-AASR------------------CEADHLQVTVAG-
TaCLE34d.2   1 ----------------------MP-ALCAV----------------------------LILLLLT--AVSR------------------CEANLLQVTVGG-
```

Central variable domain

```
TaCLE1a     61 RSLDPSS----------------------AAGAPQDQHHHHHQHHHHHRAGHHHRQRHHRLPSKWNWQRVPPSAAPGD--GEE-VDPRYGVE-------
TaCLE1d     61 QSLDPSS----------------------AAGAPQDQHHHH--HHHHHRGGHHHRQRHHRLPSKWNWQRVPPSAAPGD--GQE-IDPRYGVE-------
TaCLE1b     59 RSLDPSS----------------------AAGAPQDQQRHH--HHHHHRGGRHQRQRHHRLPSKWNWQRLPPSAAPGD--GEE-VDPRYGVE-------
TaCLE15a    42 RGATPT------------------------GRTAST-----------------------------TTTTTATRAGG--AAA---AALGDS--------
TaCLE15b    42 RGPTPT------------------------GRPAST-----------------------------TTTTTATRAGG--AAA---AALGDS--------
TaCLE15d    42 RGATPT------------------------GRTAS------------------------------TTTTTATRAGG--AAADAAAALGDS--------
TaCLE28b    51 TAAAAAR----------------------KRGGTVLQ----------------------------TGPPAAACTGE--AGG---AAYDES--------
TaCLE28d    51 TAAAAAR----------------------KRGRTVLQ----------------------------TGPPAAACAGE--AGG---AAYDES--------
TaCLE3b     40 --AALDG----------------------APPPAKGYFAEQA-----------------------SSSTARQHTARV--YRR----MHRVS--------
TaCLE3d     40 --ALVGG----------------------GPPPAKAYSSEQA-----------------------SLSTARQHTARV--YRR----MHTVS--------
TaCLE3a     42 --ALVGG----------------------GPPPAKAYSSEQS-----------------------SSSTSREHTARV--YRR----MHRVS--------
TaCLE30b    46 --ELITG----------------------QHELPPTISPTQE-----------------------AGGDDVAAADDI--GAG----KFTVS--------
TaCLE30d    44 --ELIKG----------------------QHELPPTISPTQE-----------------------AGGDDVAAADDI--GAG----KFTVS--------
TaCLE30a    45 --ELIKG----------------------QHELPPTISPTQQ-----------------------AGGDDVAAANDI--GAG----KFTVS--------
TaCLE5a     69 RRALRGG----------------------ARHRHH-RHHHR-------------------------QGAPAVPLPPP--GRDEVDMRYGVS--------
TaCLE5d     73 RRVLRGG----------------------ARHRHGGHHHHH-------------------------QGAPTVPLPPP--GRDEVDMRYGVS--------
TaCLE5b     72 RRALRGG----------------------ARHRHGGHHRHHQ------------------------QQGAPAVPLPPP--GRDEVDMRYGVS--------
TaCLE2a     36 RTGVLAP----------------------VPAEQWRQERRR-------------------------TVGRQTRGGRATT-IAPFAPRRFG----GFFRDD
TaCLE2d     36 RTGVLAP----------------------VQAEQWRQERRR-------------------------TAGRQARGGRATT-IAPFAPRRFG----GFFRDD
TaCLE2b     35 RAGVLAP----------------------VQAEQWRQQRRR-------------------------TAG-QAQGGRAT--IAPFAPRRFGGAAGGFFRDD
TaCLE20a    45 AARSASV----------------------VVSGSAHGKTPE-------------------------GAALAAARPRSG---AGGG--VFG--------DD
TaCLE20d    45 AARRAS-----------------------VVAGSAHVKTSE-------------------------GAALAAARNRNG---AGVG--GFG--------DD
TaCLE20b    39 ATRSAS------------------------VSDSAHGKTSE-------------------------GAALAAARFRSG---ASVG--GFG--------DD
TaCLE14b    45 QPRRLVE----------------------AGGNGAPAASLD-------------------------ASGKPVAAAGSS---PPPT--VFDADR-----MS
TaCLE14d    45 QPQRLVE----------------------AGGNGAPAASLD-------------------------ASGKPVAAAGPS---PPPT--VFDADR-----MS
TaCLE14a    45 QPRRLVE----------------------AGGNGAPVASFD-------------------------ASGKPVAAAGPS---PPPT--MFDADR-----MS
TaCLE21b    33 GSTFLLA----------------------V--DAARTSAFM-------------------------AMA-PLPAVAMA---PSPS--GLKDD--------
TaCLE21d    33 GSTFLLA----------------------V--DAARTSAFM-------------------------AMA-PLPAVAMA---PSPS--GLKDD--------
TaCLE21a    33 GSTFLLA----------------------V--DAARTSAFM-------------------------AMA-PLPAVAMA---PSPS--GLKDD--------
TaCLE16a    45 ASVSASG-------------------NEDGGDGPRRSAFD--------------------------ALVEGLVSIGLG--RRWRAGDGAGLVD-----GD
TaCLE16d    44 AMESAS---------------------IDGGDGPRRSAFD--------------------------VLVEGLVSIGLG--RRWRAGDGVGLVD-----GD
TaCLE16b    43 ASVSAD---------------------DSGGDGPRRSAFD--------------------------VLVEGLVSIGLG--RRWRAGDGVGLVD-----GD
TaCLE27a    44 RGRAVV-------------------------ATAIFDAAR--------------------------CKEQRKKKAGG---AAAWARDGDED-------DD
TaCLE27d    44 RGRAVV-------------------------ATATFDAAR--------------------------CKEQRKKKAGG---AVAWAGYGDEDED-----DD
TaCLE27b    44 RGRAVV-------------------------ATASFDAAR--------------------------CKEQRKKKAGG---AAAWAGDGDEDED-----DD
TaCLE33b    43 RGRAAQRWPSMPVHHAVPKPSPRAGARAVAFDATATAAAAR-------------------------CNSKSKTSAAWKRKPTAGGRDDAACDE-----DD
TaCLE4b     48 RARGRVTMMSSP--------------AQKSGGGQAGEDAYR-------------------------SSKRRIPKGPDP---------IHN----------
TaCLE4d     48 RARGGVT-------------------AQKSGGGQAGEDAYR-------------------------SSKRRIPKGPDP---------IHN----------
TaCLE4a     48 QPGGRVSTKSSP--------------VQKPGGGQAGEDAYR-------------------------SSKRRIPKGPDP---------IHN----------
TaCLE22a    47 AAAQAATLAAAPP--------VAAAATTPPSEGAALEDPYK-------------------------NSKRKVPNGPDP---------IHN----------
TaCLE22d    48 AAAQAATLAAAPP--------VAAAAMAPPSERAALEDPYK-------------------------NSKRKVPNGPDP---------IHN----------
TaCLE25a    47 AGDGGETR------------------MTLEDFR--ADDPFQ-------------------------DSARRVPNGPDP---------IHN----------
TaCLE25d    49 PGDGGEAR------------------RTLEDFR--ADDPFQ-------------------------DSARRVPNGPDP---------IHN----------
TaCLE25b    47 AGDGGEAR------------------RTLENFR--ADDPFQ-------------------------DSARRVPNGPDP---------IHN----------
TaCLE29b    41 DAAAGGRR------------------ALVPAAR---SGRFR-------------------------PRPRRW-NSAGL---------VDS----------
TaCLE29d    47 PAAASSSF------------------PVAAGGRRAFAGPGR-------------------------SRARRW-NSAGL---------ADS----------
TaCLE6a     26 PRLIQHPA----------------------VALAHAAANGK--------------------------PAGVPPSKWNMPRTLG---AV--------
TaCLE6d     26 ARLIQHPA----------------------VALAHTAANGK--------------------------PAGVPPAKWNMRRTLGA--AV--------
TaCLE17a    27 ARAVAGVV----------------------YAAAVANAAGA-------------------------AAATSGAGVPPGRWN-ARRLQGDAAH--------
TaCLE17b    27 ARAVAGVV----------------------YAAAVTNAAGA-------------------------AAATGGAGVPPGRWN-ARRLQGDAAH--------
TaCLE17d    27 ARAVAHVL----------------------YAAAVTNAVGA-------------------------AS---GAGVRPGRWNTARRLQGDAAH--------
TaCLE18a    27 ARAVPDVV----------------------YAAAAG-------------------------------GGIRRGRWNSARRLEGDAAR--------
TaCLE18d    27 ARAVPDVV----------------------YSAAAGNAA----------------------------GGGIRRGRWNSARRLEGDAAR--------
TaCLE18b    27 ARAVADVV----------------------YAAAATNAA----------------------------AGGIRRGKWNSARRLEGDAAR--------
TaCLE13b    36 SRSAAAAL----------------------QRVEMAAMYTP-------------------------QDLQEKPDVTKDAEEDVSTTGFGAEE--------
TaCLE13d    36 SRSAAAAL----------------------QRVEMAAVYTP-------------------------QDLQEKPDVTKDAEEDVSTTGFGAEE--------
TaCLE13a    38 SRAAAAAL----------------------QRVEMAAMYTP-------------------------QDLQEKPDVTKDAEEDVSTTGFGAEE--------
TaCLE32b    40 GGGGGGNA----------------------LQHGLVDGHRP-------------------------RHVASFTTGDDVVEAPSSQAAGGDGA--------
TaCLE32d    40 GGGGGGNA----------------------LQHGLVD-------------------------------------DAPSSQAAGGDDA-------
TaCLE32a    40 DGGR--NA----------------------LQHGFVDGHRP-------------------------RPVASLTMGDDVVEAPSSQAAGGDGA--------
TaCLE7a.1   38 ---REAT------------------------VAVMPMAGG--------------------------HFARKVLREEMVQADGD---VDIAGS--------
TaCLE7b.1   38 ---RGAT------------------------VAATTMARG--------------------------RFARKVLREEMVQADGD---VDIAGS--------
TaCLE7a.2   38 ---REAT------------------------VAPTTMARG--------------------------RFVRKVLREEMVQADDN-DVVDIAGS--------
TaCLE7b.2   38 ---REAT------------------------VAPTMMARG--------------------------RFVRKVLREEMVQADDD-GVLDIGGS--------
TaCLE7d     38 GGRQWGT------------------------VAPTMLTRG--------------------------RFVRKVLREEMVQADDD-GVVDIGGS--------
TaCLE8b.1   38 TN--KAA------------------------VAAMTVATG--------------------------RFVRKVLREEVIEVDDD-GVVDIGGS--------
TaCLE8b.2   38 T---KAT------------------------VAATTMATG--------------------------RFVRKLLREEVVEVDDE-GVVDIGGS--------
TaCLE8a     38 R---KAT------------------------VAATTMAAG--------------------------RFVRNVLREEVVQADDVNGVVDIGDS--------
TaCLE8d     38 R---KAT------------------------VTASTMATG--------------------------HLVRKVLREEVVQANND-GVVHIGGS--------
TaCLE11b    38 R---KAT------------------------MAATTMATK--------------------------RLVRKLLQEEVVQADDD-GVIDIGGS--------
TaCLE11d    40 R---KAT------------------------VAATAMATR--------------------------RFVRKLLREEVVQADDD-GVVDIGGS--------
TaCLE9a     38 R---EAT-----------------------APAAMAMARG--------------------------PFVRKLLREEMVKADGQ---GDIGQS--------
TaCLE9d     38 R---EAT-----------------------APGAMAMARG--------------------------PFVRKLLREEMVKADGQ---GDIGQS--------
TaCLE12b    38 R---EAT-----------------------APAAMAMARG--------------------------RFVMKVLREEMVQADGQ---GDIGQS--------
TaCLE12d    38 R---EAT-----------------------APAAMAMAAGG-------------------------RFVRKVLREEMVQAGRQ---GDIGQS--------
TaCLE10b    38 R---EAT-----------------------APAAMAMATG--------------------------RFVRKVLREEMVQADGH---GDIGQS--------
TaCLE10a    38 R---EAT-----------------------APAAMATAKG--------------------------RFVRMVLREEMLQANGQ---GGIGES--------
TaCLE10d    33 R---EATA----------------------APAAMAMARG--------------------------RFVTKVLREEMVQANGQ---GGIGES--------
TaCLE19a    31 ----EAP---------------------------SATGGTG---------------------------VTEVSMKVPDEGQRQ--VGKAAES--------
TaCLE19b    31 ----EAP---------------------------SATGGTG---------------------------VTEVSMKVPDEGQRQ--VGKAAES--------
TaCLE19d    31 ----EAP---------------------------SATDGAG---------------------------VTEVSMKVPGEGQRQ--VGTAAES--------
TaCLE23a    32 RRQLQDV------------------------VVRLQA-----------------------------ASTAVAAR---PRQEGIADPVYETS--------
TaCLE23d    32 RRQLQDV------------------------VVRLQATAA--------------------------ASTAAAAR---PRQEGIADPVYGTS--------
TaCLE23b    32 RRQLHDV------------------------VVRLQATAA--------------------------ASTAAAVG---PRQE-IADPVYGTS--------
TaCLE24a    35 RRQLEDVD-----------------------RGG----PAV-------------------------ASTTAAVR---PPQEIADLVVYGTS--------
TaCLE24d    36 RRQLEDVD-----------------------GGGGLQATAT-------------------------ASTTAAVR---PPQEIADLVVYGTS--------
TaCLE24b    35 RRQLEDVD-----------------------SGGGLQATAV-------------------------ASTTAAVR---PQQEITDLVVYGTS--------
TaCLE31a    32 RRCLQDTV-----------------------VIGGGPTPTG-------------------------ASTTGTL----PRDAAPDISVD-RS--------
TaCLE31d    32 RRCLQDAV-----------------------VIGGSPTPTG-------------------------ASTTGTL----PRDAEPDISVD-RS--------
TaCLE31b    32 RRWLQDAV-----------------------VIGGSPTTTA-------------------------ASTTGTL----PRDAEPDISVD-RS--------
TaCLE34a    33 RRMLAGGS-----------------------NAAAAFSRPT-------------------------ETTAGSSSWRAADGRATAAMPYSES--------
TaCLE34d.1  34 RRMLAGGS-----------------------N-AAALSRQV--------------------------ETTAASSTWRAAAGRATAAMPYSES--------
TaCLE35a    32 RRMLAGGS-----------------------NVAAVFSREA-------------------------ATTVASSARQSAAGR--AAMPYSES--------
TaCLE35b.1  32 RRMLAGGS-----------------------NACTIFSREA--------------------------ATTVASSARRSAAGR--AAMPYSES--------
TaCLE35b.2  32 RRMLAGGS-----------------------NAAAVFSREA--------------------------ETTVASSARRSAAGR--AAMPYSES--------
TaCLE35d    32 RRMLAGGS-----------------------NAATVYSREA-------------------------ATAVANSARRSADGR--AAMPYSES--------
TaCLE34d.2  31 RRMLAGGS-----------------------NAAAVFSRPA--------------------------ETTAASTMRWAAAGRATAAMPYSES--------
```

CLE domain

```
TaCLE1a     128 KRLVPTGPNPLHH-----------
TaCLE1d     126 KRLVPTGPNPLHH-----------
TaCLE1b     124 KRLVPTGPNPLHH-----------
TaCLE15a     74 KRLVPQGPNPLHN-----------
TaCLE15b     74 KRLVPQGPNPLHN-----------
TaCLE15d     76 KRLVPQGPNPLHN-----------
TaCLE28b     86 KRMVPQGPNPLHN-----------
TaCLE28d     86 KRMVPQGPNPLHN-----------
TaCLE3b      78 KRLVPQGPNPLHN-----------
TaCLE3d      78 KRLVPQGPNPLHN-----------
TaCLE3a      80 KRLVPQGPNPLHN-----------
TaCLE30b     84 RRAVPQGPNPLHN-----------
TaCLE30d     82 RRAVPQGPNPLHN-----------
TaCLE30a     83 RRAVPQGPNPLHN-----------
TaCLE5a     110 KRRVPTGPNPLHN-----------
TaCLE5d     115 KRRVPTGPNPLHN-----------
TaCLE5b     116 KRRVPTGPNPLHN-----------
TaCLE2a      84 KRFAPTGSNPLHNL----------
TaCLE2d      84 KRFAPTGSNPLHNL----------
TaCLE2b      85 KRFAPTGSNPLHNL----------
TaCLE20a     85 KRMAPSGSNPLHNRR---------
TaCLE20d     84 KRMAPSGSNPLHNLR---------
TaCLE20b     77 KRMAPSGSNPLHNLR---------
TaCLE14b     88 KRRVRRGSDPIHNKC---------
TaCLE14d     88 KRRVRRGSDPIHNKC---------
TaCLE14a     88 KRRVRRGSDPIHNKC---------
TaCLE21b     70 KRRVPTGANPLHNR----------
TaCLE21d     70 KRRVPTGANPLHNR----------
TaCLE21a     70 KRRVPTGANPLHNR----------
TaCLE16a     93 KRRVPTGPNPLHNR----------
TaCLE16d     90 KRRVPTGPNPLHNR----------
TaCLE16b     89 KRRVPTGPNPLHNR----------
TaCLE27a     83 KHVVPTGPNPLHNR----------
TaCLE27d     85 KHVVPTGPNPLHNR----------
TaCLE27b     85 KHVVPTGPNPLHNR----------
TaCLE33b    113 KRRIPTGPNPLHNR----------
TaCLE4b      90 RRAGKTTIAPRRRD----------
TaCLE4d      85 RRAGKTTIAPRRRD----------
TaCLE4a      90 RRAGKTTIAPRRRD----------
TaCLE22a     95 RRARWGDAPARRV-----------
TaCLE22d     96 RRARWGDAPARRV-----------
TaCLE25a     83 RGAGKSGRSPGRE-----------
TaCLE25d     85 RGAGKSGRSPGRE-----------
TaCLE25b     83 RGAGKSGRSPGRA-----------
TaCLE29b     75 KHEVPSGPNPDSNR----------
TaCLE29d     84 KHEVPSGPNPDSNR----------
TaCLE6a      63 KRTVPGGPDPQHHY----------
TaCLE6d      64 KRTVPGGPDPQHHY----------
TaCLE17a     71 KREVPGGPDPEHHH----------
TaCLE17b     71 KREVPGGPDPEHHH----------
TaCLE17d     69 KREVPGGPDPEHHH----------
TaCLE18a     61 KREVPGGPDPQHHH----------
TaCLE18d     65 KREVPGGPDPQHHH----------
TaCLE18b     65 KREVPGGPDPQHHH----------
TaCLE13b     81 EREVPTGPDPIHHHGREPRRRQSP
TaCLE13d     81 EREVPTGPDPIHHHGRGPRRRQSP
TaCLE13a     83 EREVPTGPDPIHHHGRGPRRRQSP
TaCLE32b     85 KREVPGGPDPIHHHGSVPPTSVAP
TaCLE32d     68 DREVPGGPDPIHHHGSVPPTSVAP
TaCLE32a     83 KREVPGGPDPIHHHGSIPPNSVAP
TaCLE7a.1    74 KRKSPGGPDPQHH-----------
TaCLE7b.1    74 KRKSPGGPDPQHH-----------
TaCLE7a.2    76 KRKSPGGPDPQHH-----------
TaCLE7b.2    76 KRKSPGGPDPQHH-----------
TaCLE7d      79 KRKSPGGPDPQHH-----------
TaCLE8b.1    77 KRKSPGGPDPQHH-----------
TaCLE8b.2    76 KRKSPGGPDPQHH-----------
TaCLE8a      77 KRKSPGGPDPQHH-----------
TaCLE8d      76 KRKSPGGPDPQHH-----------
TaCLE11b     76 KRKSPGGPDPQHH-----------
TaCLE11d     78 KRKSPGGPDPQHH-----------
TaCLE9a      75 KRTSPGGPDPQHH-----------
TaCLE9d      75 KRTSPGGPDPQHH-----------
TaCLE12b     75 KRTSPGGPDPQHH-----------
TaCLE12d     76 KRTSPGGPDPQHH-----------
TaCLE10b     75 KRTSPGGPDPQHH-----------
TaCLE10a     75 KRTSPGGPDPQHH-----------
TaCLE10d     71 KRTSPGGPDPQHH-----------
TaCLE19a     63 KRLSPGGSDPQHH-----------
TaCLE19b     63 KRLSPGGSDPQHH-----------
TaCLE19d     63 KRLSPGGSDPQHH-----------
TaCLE23a     67 KRLSPGGSNPQHHR----------
TaCLE23d     70 KRLSPGGSNPQHHR----------
TaCLE23b     69 KRVSPGGSNPQHHR----------
TaCLE24a     71 KRLSPGGSNPQHHH----------
TaCLE24d     76 KRLSPGGSNPQHHH----------
TaCLE24b     75 KRLSPGGSNPQHHH----------
TaCLE31a     70 KRLSPGGSNPQHH-----------
TaCLE31d     70 KRLSPGGSNPQHH-----------
TaCLE31b     70 KRLSPGGSNPQHH-----------
TaCLE34a     76 KRSSPGGPDPQHH-----------
TaCLE34d.1   76 KRSSPGGPDPQHH-----------
TaCLE35a     73 KRSSPGGPDPQHH-----------
TaCLE35b.1   73 KRSSPGGPDPQHH-----------
TaCLE35b.2   73 KRSSPGGPDPQHH-----------
TaCLE35d     73 KRSSPGGPDPQHH-----------
TaCLE34d.2   74 KRSSPGGPDPQHH-----------
consensus   201 kr  pggp p hh
```
