# Supplementary material for: Identification and Functional Analysis of the CLAVATA3/EMBRYO SURROUNDING REGION (CLE) Gene Family in Wheat
Source: Int J Mol Sci. 2019 Sep 3;20(17):4319. doi: 10.3390/ijms20174319 (PMC6747155; doi:10.3390/ijms20174319)
Supplement: Supplementary file 1 [file ijms-20-04319-s001.zip › Supplementary Figure S2-S6.pdf]

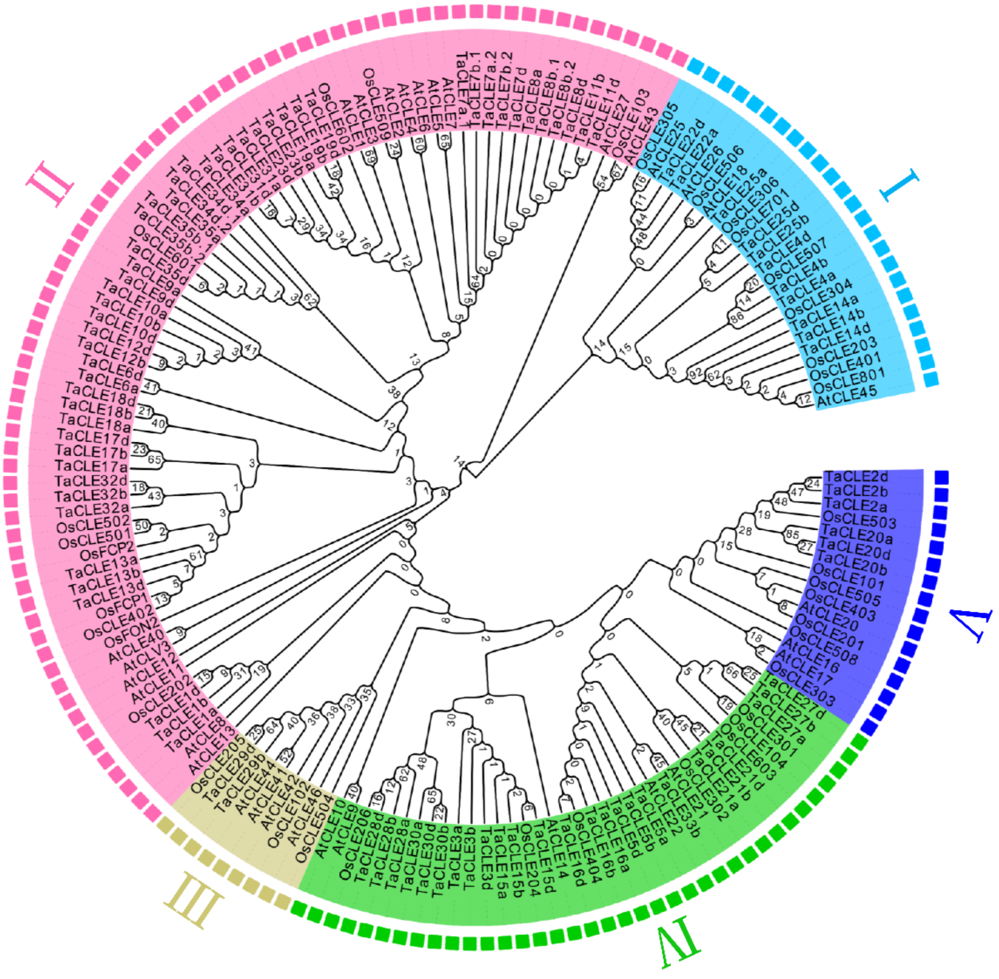

Supplementary Figure S2 Phylogenetic analysis was performed using the multiple sequence alignment generated with entire mature peptide sequences.

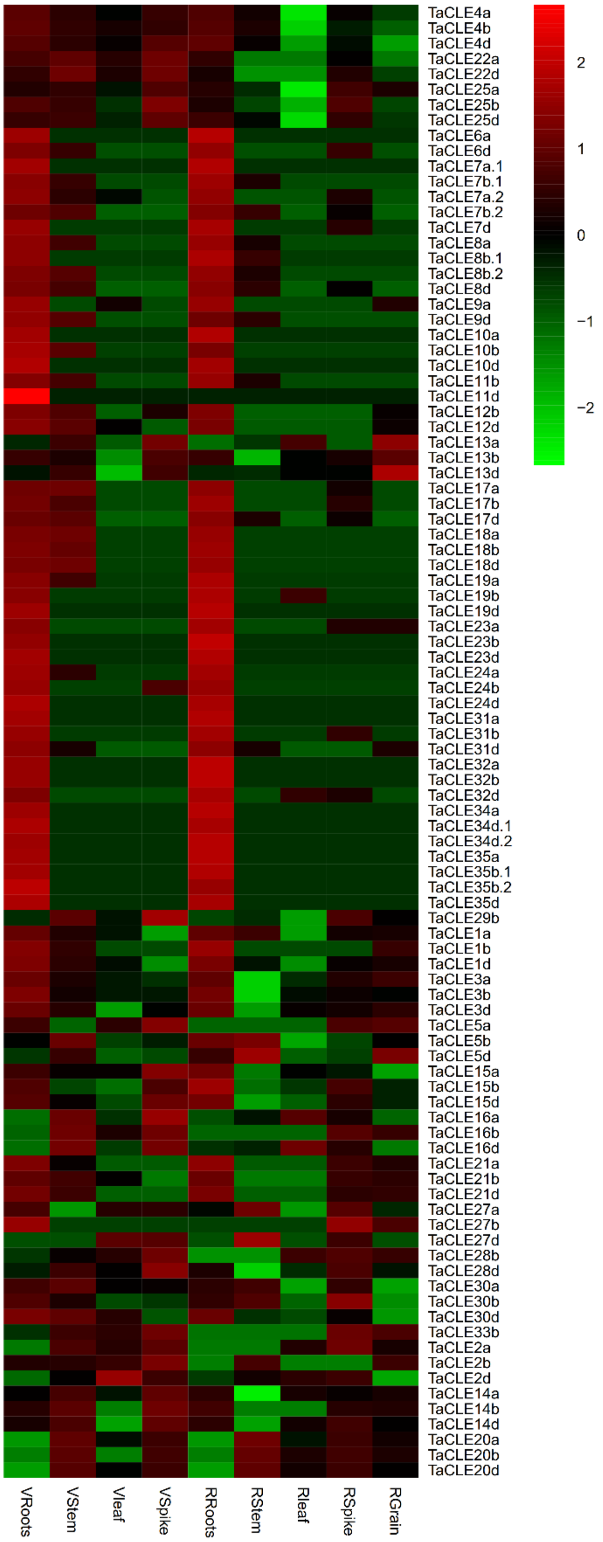

Supplementary Figure S3 *In silico* expression profiling of TaCLE genes in different organs at different growth stages of Chinese Spring wheat. The expression data were generated from the expVIP database (http://www.wheat-expression.com/). The color scale at the top represents the expression values: green indicates low levels of transcript abundance, and red indicates high levels. VRoots: roots in vegetative stage, VStem: stem in vegetative stage, Vleaf: leaf in vegetative stage, VSpike: Spike in vegetative

stage. RRoots: roots in reproductive stage, RStem: stem in reproductive stage, Rleaf: leaf in reproductive stage, RSpike: Spike in reproductive stage. RGrain: grain in reproductive stage.

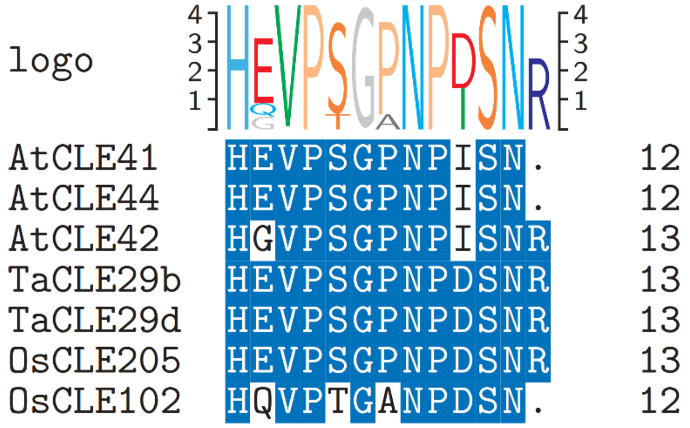

Supplementary Figure S4: Multiple sequence alignment of the CLE domains of TDIFs in wheat, *Arabidopsis* and rice

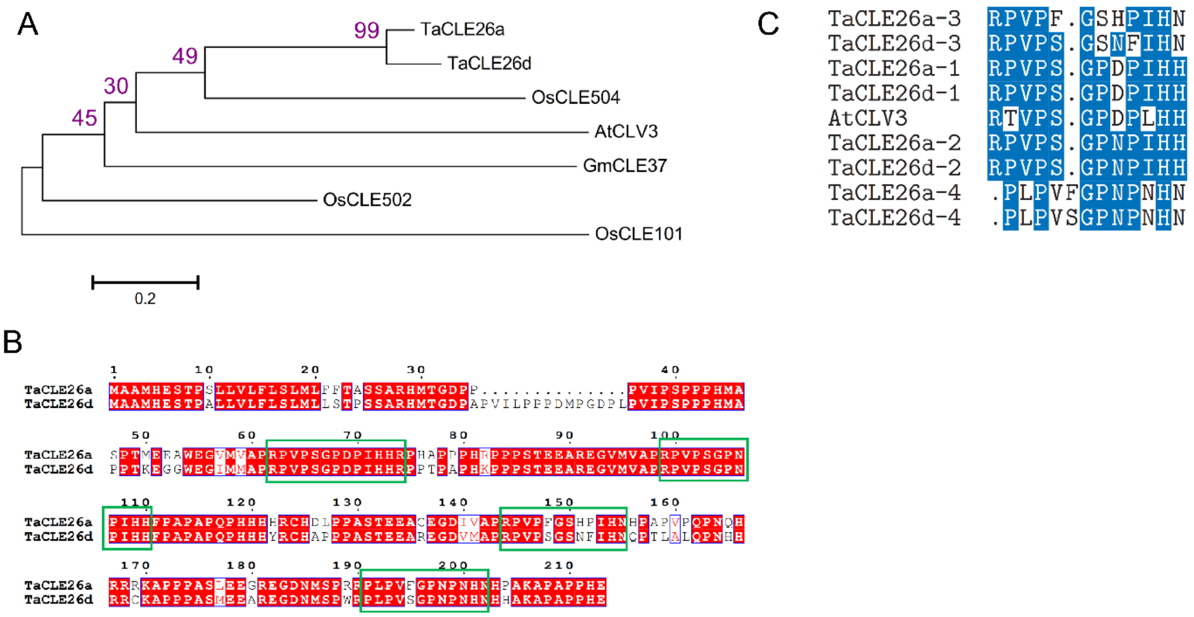

Supplementary Figure S5: The identified multi-CLE peptide-encoding genes in wheat. (A) Phylogenetic analysis of multi-CLE peptide-encoding genes. (B) Multiple sequence alignment of TaCLE26a and TaCLE26d, with putative 12 amino acid residue CLE domains highlighted by a green box. (C) Multiple sequence alignment of the CLE domain of AtCLV3 and four putative CLE domains of TaCLE26a and TaCLE26d.

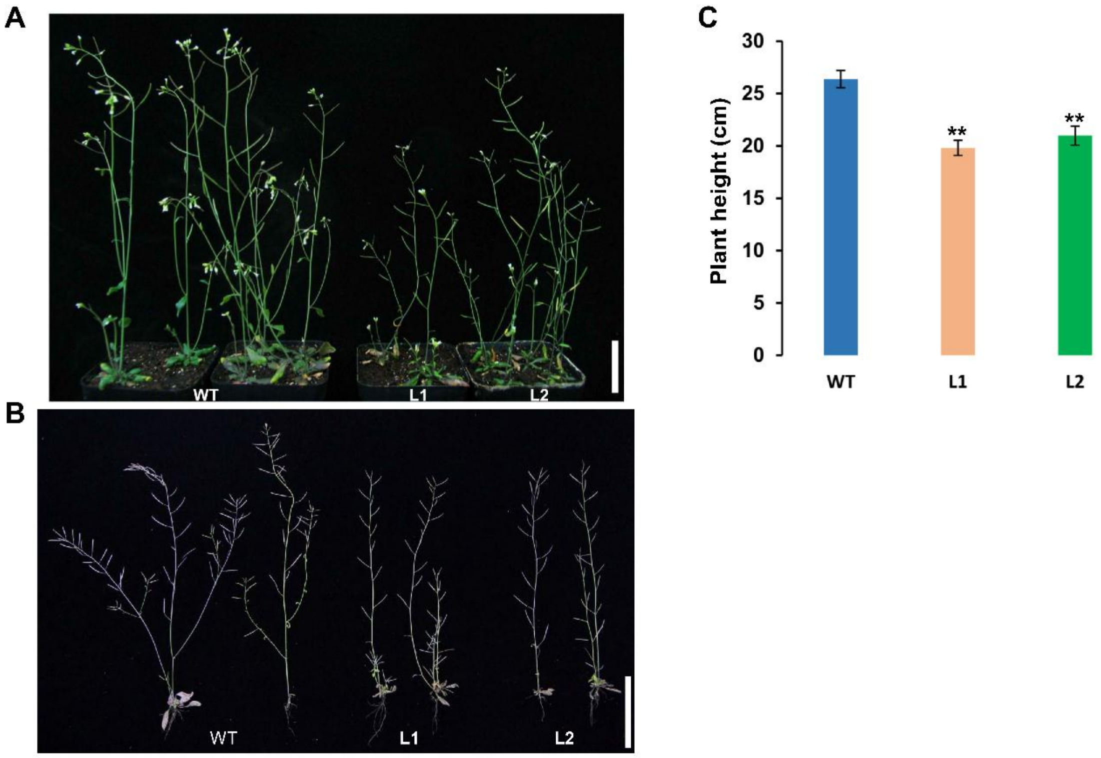

Supplementary Figure S6: Phenotypes of TaCLE3d-OE. (A) 35-day-old plants. (B) 60-day-old plants. (C) Quantification of the primary stem length of 60-day-old plants. The bar graphs indicate the mean ± SE. Statistical significance (Student's t-test) compared with wild *Arabidopsis* (WT) is indicated: **P<0.01. Scale bars: 3cm in A, 8 cm in B.
