# Supplementary material for: Identification and Functional Analysis of the CLAVATA3/EMBRYO SURROUNDING REGION (CLE) Gene Family in Wheat
Source: Int J Mol Sci. 2019 Sep 3;20(17):4319. doi: 10.3390/ijms20174319 (PMC6747155; doi:10.3390/ijms20174319)
Supplement: Supplementary file 1 [file ijms-20-04319-s001.zip › Supplementary Table S1.docx]

**Supplementary Table S3.** *Features of the wheat (Triticum aestivum L.) CLE genes*

| **Name** | **Chromosome location** | **Orientation** | **Pre**  **-propeptide**  **length^a^** | **Predicted**  **Intron** | | **SP**  **cleavage**  **site^b^** | |
| --- | --- | --- | --- | --- | --- | --- | --- |
| TaCLE1a | 1A:24077190:24078148 | Forward | 140 | N | 24 | |  |
| TaCLE1b | 1B:39311181:39311591 | Forward | 136 | N | 24 | |  |
| TaCLE1d | 1D:22902616:22903961 | Forward | 138 | N | 24 | |  |
| TaCLE2a | 1A:118877092:118877385 | Reverse | 97 | N | 19 | |  |
| TaCLE2b | 1B:171200783:171201079 | Reverse | 98 | N | 19 | |  |
| TaCLE2d | 1D:111951371:111952399 | Reverse | 97 | Y | 19 | |  |
| TaCLE3a | 1A:308810895:308811509 | Reverse | 92 | N | 33 | |  |
| TaCLE3b | 1B:342122341:342123053 | Forward | 90 | N | 31 | |  |
| TaCLE3d | 1D:236808855:236809127 | Forward | 90 | N | 31 | |  |
| TaCLE4a | 1A:514537317:514538874 | Reverse | 103 | Y | - | |  |
| TaCLE4b | 1B:563996095:563997859 | Reverse | 103 | Y | - | |  |
| TaCLE4d | 1D:417366373:417367571 | Reverse | 98 | Y | - | |  |
| TaCLE5a | 1A:520793444:520793812 | Reverse | 122 | N | 24 | |  |
| TaCLE5b | 1B:574267312:574268267 | Reverse | 128 | N | 24 | |  |
| TaCLE5d | 1D:424991537:424992317 | Reverse | 127 | N | 24 | |  |
| TaCLE6a | 1A:521907397:521907627 | Forward | 77 | N | 23 | |  |
| TaCLE6d | 1D:426920281:426920514 | Forward | 77 | N | 23 | |  |
| TaCLE7a.1 | 1A:559800433:559800693 | Forward | 86 | N | 28 | |  |
| TaCLE7b.1 | 1B:645280128:645280388 | Forward | 86 | N | 28 | |  |
| TaCLE7a.2 | 1A:559841631:559841983 | Forward | 88 | N | 28 | |  |
| TaCLE7b.2 | 1B:645308072:645308350 | Forward | 88 | N | 28 | |  |
| TaCLE7d | 1D:467487520:467491489 | Forward | 91 | Y | 28 | |  |
| TaCLE8a | 1A:559836875:559837550 | Forward | 89 | N | 27 | |  |
| TaCLE8b.1 | 1B:645275021:645275290 | Reverse | 89 | N | 30 | |  |
| TaCLE8b.2 | 1B:645303652:645303918 | Forward | 88 | N | 28 | |  |
| TaCLE8d | 1D:467467355:467467621 | Forward | 88 | N | 28 | |  |
| TaCLE9a | 1A:559881495:559881758 | Forward | 87 | N | 26 | |  |
| TaCLE9d | 1D:467510704:467510967 | Forward | 87 | N | 30 | |  |
| TaCLE10a | 1A:560486475:560486738 | Reverse | 87 | N | 26 | |  |
| TaCLE10b | 1B:645340575:645340838 | Forward | 87 | N | 26 | |  |
| TaCLE10d | 1D:467501594:467501915 | Forward | 83 | N | 25 | |  |
| TaCLE11b | 1B:645294141:645294407 | Forward | 88 | N | 28 | |  |
| TaCLE11d | 1D:467471899:467472171 | Forward | 90 | N | 30 | |  |
| TaCLE12b | 1B:645319905:645320168 | Forward | 87 | N | 26 | |  |
| TaCLE12d | 1D:467494685:467494951 | Forward | 88 | N | 26 | |  |
| TaCLE13a | 2A:561552087-561552404 | Forward | 106 | N | 32 | |  |
| TaCLE13b | 2B:503774662-503774979 | Reverse | 104 | N | 32 | |  |
| TaCLE13d | 2D:426946740-426947055 | Reverse | 104 | N | 32 | |  |
| TaCLE14a | 2A:573366983:573367291 | Reverse | 102 | N | 22 | |  |
| TaCLE14b | 2B:488868817:488869125 | Forward | 103 | N | 22 | |  |

**Table 1.** *Continued*

| **Name** | **Chromosome location** | **Orientation** | **Pre**  **-propeptide**  **length^a^** | **Predicted**  **Intron** | | **SP**  **cleavage**  **site^b^** | |
| --- | --- | --- | --- | --- | --- | --- | --- |
| TaCLE14d | 2D:416861673:416861984 | Forward | 103 | N | 21 | |  |
| TaCLE15a | 2A:614384466:614385129 | Forward | 86 | Y | 21 | |  |
| TaCLE15b | 2B:552028724:552032421 | Forward | 86 | Y | 30 | |  |
| TaCLE15d | 2D:472860703:472861362 | Forward | 88 | N | 30 | |  |
| TaCLE16a | 2A:729346578:729347592 | Forward | 106 | N | 28 | |  |
| TaCLE16b | 2B:722862712:722863020 | Forward | 102 | N | 27 | |  |
| TaCLE16d | 2D:595211811:595213105 | Forward | 103 | Y | 28 | |  |
| TaCLE17a | 3A:523608479:523609314 | Reverse | 84 | N | 28 | |  |
| TaCLE17b | 3B:528425285:528425539 | Reverse | 84 | N | 28 | |  |
| TaCLE17d | 3D:402747288:402747536 | Reverse | 82 | N | 23 | |  |
| TaCLE18a | 3A:523727432:523727656 | Reverse | 74 | N | 19 | |  |
| TaCLE18b | 3B:528492880:528493116 | Reverse | 78 | N | 19 | |  |
| TaCLE18d | 3D:402774829:402775065 | Reverse | 78 | N | 19 | |  |
| TaCLE19a | 3A:523746098:523746325 | Reverse | 75 | N | 21 | |  |
| TaCLE19b | 3B:528631082:528631309 | Reverse | 76 | N | 21 | |  |
| TaCLE19d | 3D:402796032:402796259 | Reverse | 75 | N | 26 | |  |
| TaCLE20a | 3A:57812878:57813177 | Forward | 99 | N | 25 | |  |
| TaCLE20b | 3B:71804039:71804977 | Reverse | 91 | Y | 23 | |  |
| TaCLE20d | 3D:46197037:46197848 | Forward | 98 | N | 25 | |  |
| TaCLE21a | 4A:91312751:91313002 | Reverse | 83 | N | - | |  |
| TaCLE21b | 4B:458953240:458953491 | Forward | 83 | N | - | |  |
| TaCLE21d | 4D:373583742:373583993 | Forward | 83 | N | - | |  |
| TaCLE22a | 4A:589472934:589473664 | Forward | 107 | Y | 29 | |  |
| TaCLE22b | 4B:23705749:23705837 | -- | -- | -- | -- | |  |
| TaCLE22d | 4D:13096296:13097429 | Reverse | 108 | Y | 32 | |  |
| TaCLE23b | 4B:613714927:613715175 | Forward | 83 | N | 21 | |  |
| TaCLE23d | 4D:483448210:483448461 | Reverse | 83 | N | 25 | |  |
| TaCLE24a | 5A:605143707:605143973 | Reverse | 84 | N | 19 | |  |
| TaCLE24b | 5B:594759703:594759969 | Reverse | 88 | N | 24 | |  |
| TaCLE24d | 5D:483658676:483658945 | Reverse | 89 | N | 24 | |  |
| TaCLE25a | 5A:632663821:632665116 | Forward | 95 | Y | 32 | |  |
| TaCLE25b | 5B:634981481:634983144 | Forward | 95 | Y | 32 | |  |
| TaCLE25d | 5D:505123734:505125051 | Forward | 98 | Y | 34 | |  |
| TaCLE26a | 5A:645976978:645977619 | Forward | 213 | N | 28 | |  |
| TaCLE26b | 5B:653782454:653782698 | Forward | -- | -- | - | |  |
| TaCLE26d | 5D:519407592:519408596 | Forward | 227 | Y | 28 | |  |
| TaCLE23a | 5A:663385801:663386043 | Reverse | 80 | N | 21 | |  |
| TaCLE27a | 6A:92852991:92853733 | Reverse | 96 | N | 29 | |  |
| TaCLE27b | 6B:150151626:150151922 | Reverse | 98 | N | 29 | |  |
| TaCLE27d | 6D:77524678:77524974 | Reverse | 98 | N | 29 | |  |

**Table 1.** *Continued*

| **Name** | **Chromosome location** | **Orientation** | **Pre**  **-propeptide**  **length^a^** | **Predicted**  **Intron** | | **SP**  **cleavage**  **site^b^** | |
| --- | --- | --- | --- | --- | --- | --- | --- |
| TaCLE28a | 6A:445562192:445562511 | Forward | 96 | N | 36 | |  |
| TaCLE28b | 6B:476126147:476126443 | Reverse | 98 | N | 36 | |  |
| TaCLE28d | 6D:308900896:308901192 | Reverse | 98 | N | 36 | |  |
| TaTDIF29a | 6A:600773041:600773366 | Forward | - | - | - | |  |
| TaTDIF29b | 6B:691034557:691034847 | Forward | 88 | Y | 28 | |  |
| TaTDIF29d | 6D:454614870:454615192 | Reverse | 97 | N | 29 | |  |
| TaCLE30a | 6A:613338242:613338529 | Forward | 95 | N | 33 | |  |
| TaCLE30b | 6B:712244601:712248353 | Forward | 96 | N | 26 | |  |
| TaCLE30d | 6D:467042356:467043023 | Forward | 95 | N | 32 | |  |
| TaCLE31a | 7A:511054738:511054986 | Forward | 82 | N | 25 | |  |
| TaCLE31b | 7B:438991265:438991513 | Forward | 83 | N | 25 | |  |
| TaCLE31d | 7D:422109141:422109389 | Reverse | 83 | N | 25 | |  |
| TaCLE32a | 7A:511405885:511406205 | Forward | 106 | N | 25 | |  |
| TaCLE32b | 7B:439259025:439259351 | Forward | 109 | N | 25 | |  |
| TaCLE32d | 7D:421856050:421856379 | Reverse | 92 | Y | 25 | |  |
| TaCLE33b | 7B:580185901:580186281 | Reverse | 126 | N | 26 | |  |
| TaCLE33d | 7D:537683355:537683777 | Reverse | - | - | - | |  |
| TaCLE34a | 7A:69225137:69225430 | Forward | 97 | N | 32 | |  |
| TaCLE34d.1 | 7D:64893828:64894121 | Forward | 97 | N | 33 | |  |
| TaCLE34d.2 | 7D:64912484:64912771 | Forward | 96 | N | 31 | |  |
| TaCLE35a | 7A:69526946:69527203 | Forward | 85 | N | 23 | |  |
| TaCLE35b.1 | 7B:6797030:6797287 | Forward | 86 | N | 23 | |  |
| TaCLE35b.2 | 7B:6921009:6921266 | Reverse | 85 | N | 23 | |  |
| TaCLE35d | 7D:65390393:65390769 | Forward | 96 | N | 33 | |  |

*a* Number of amino acid residues.

*b* After amino acid number listed.

Listed are the genetic location, pre-propeptide length, predicted intron presence, gene orientation, and SignalP signal peptide (SP) cleavage site.
